# Aminoazole-Based Diversity-Oriented Synthesis of Heterocycles

**DOI:** 10.3389/fchem.2018.00527

**Published:** 2018-11-13

**Authors:** Maryna V. Murlykina, Alisa D. Morozova, Ievgen M. Zviagin, Yana I. Sakhno, Sergey M. Desenko, Valentyn A. Chebanov

**Affiliations:** ^1^Department of Organic and Bioorganic Chemistry, State Scientific Institution “Institute for Single Crystals”, National Academy of Sciences of Ukraine (NAS), Kharkiv, Ukraine; ^2^Chemistry Faculty, Karazin Kharkiv National University, Kharkiv, Ukraine

**Keywords:** aminoazole, diversity-oriented synthesis, heterocycle, heterocyclization reaction, multicomponent reaction, microwave-assisted organic synthesis, ultrasonication, click-chemistry

## Abstract

The comprehensive review contains the analysis of literature data concerning reactions of heterocyclization of aminoazoles and demonstrates the application of these types of transformations in diversity-oriented synthesis. The review is oriented to wide range of chemists working in the field of organic synthesis and both experimental and theoretical studies of nitrogen-containing heterocycles.

## Introduction

Heterocyclic compounds are backbone of drug design–about 80% of the known small molecule drugs belong to this type of substances and among them 60% relates to nitrogen containing heterocycles (Kombarov et al., 2010; Vitaku et al., 2014; Taylor et al., 2016). On the other hand, heterocyclic compounds play important role in other branches of science and are the base of all living organisms. Therefore, study of the appropriate field of organic chemistry is a very important challenge that has been attracting attention of numerous scientific groups for last decades and stimulating for detailed study of the topic including the search for novel and development of known synthetic methods.

**Figure 1:**
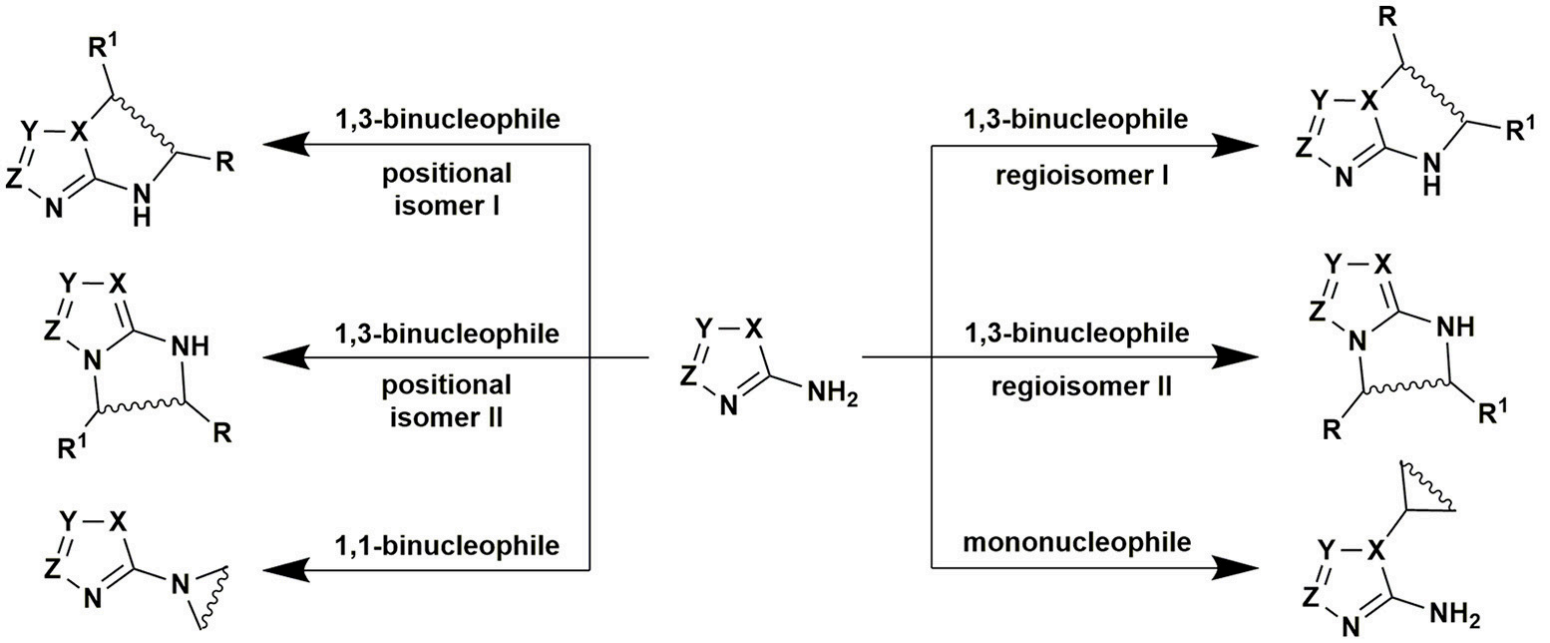
Diversity of heterocyclizations involving aminoazoles.

One of the important pathways to nitrogen containing heterocycles is reactions of aminoazoles (two-component, one-pot, multicomponent, etc.) being efficient mono-, bi- and polynucleophiles with different electrophiles. The presence of several alternative reaction centers in aminoazoles often makes them useful reagents in controlled multidirectional interactions providing the possibility to synthesize diverse chemotypes of final products (see some examples in Figure 1). Such approach is widely used in the modern heterocyclic chemistry and some books and reviews have been already published in this field (Desenko, 1995; Chebanov and Desenko, 2006, 2012, 2014; Chebanov et al., 2008a, 2010; Moderhack, 2011; Sedash et al., 2012; Tkachenko and Chebanov, 2016; Aggarwal and Kumar, 2018), however, many of them deal with particular problems of aminoazole chemistry and actually during long period no comprehensive analysis of the problem has been made.

Thus, the present review is devoted to diversity-oriented reactions of heterocyclization involving aminoazoles as a key reagent. It presents analysis of literature mainly from 2010 till present and three main types of such reactions are discussed: multicomponent reactions including application of condition-based divergence strategy for the control of their directions; two-component heterocyclizations and one-pot cascade processes; “click”-chemistry concerning azoles and aminoazoles.

## Main part

### Multicomponent reactions of aminoazoles involving cyclic CH-acids

Multicomponent reactions (MCRs) involving aminoazoles and aldehydes with cyclic CH-acids (different ketones, 1,3-diketones, Meldrum's acid etc.) are similar to the classic Hantzsch or Biginelly condensations. In early publications they had often resulted in the formation of mixtures of positional and regioisomers, therefore, some efficient methods for tuning chemo- and regioselectivity of such multicomponent heterocyclizations, including Condition-based divergence strategy to switch their directions by simple variation of the reaction conditions (solvent, temperature, method of activation–microwave irradiation (MW) and ultrasonication (US), catalyst, etc), were found and developed (Desenko, 1995; Chebanov and Desenko, 2006, 2012; Chebanov et al., 2008a, 2010; Ruijter et al., 2011).

**Figure 2:**
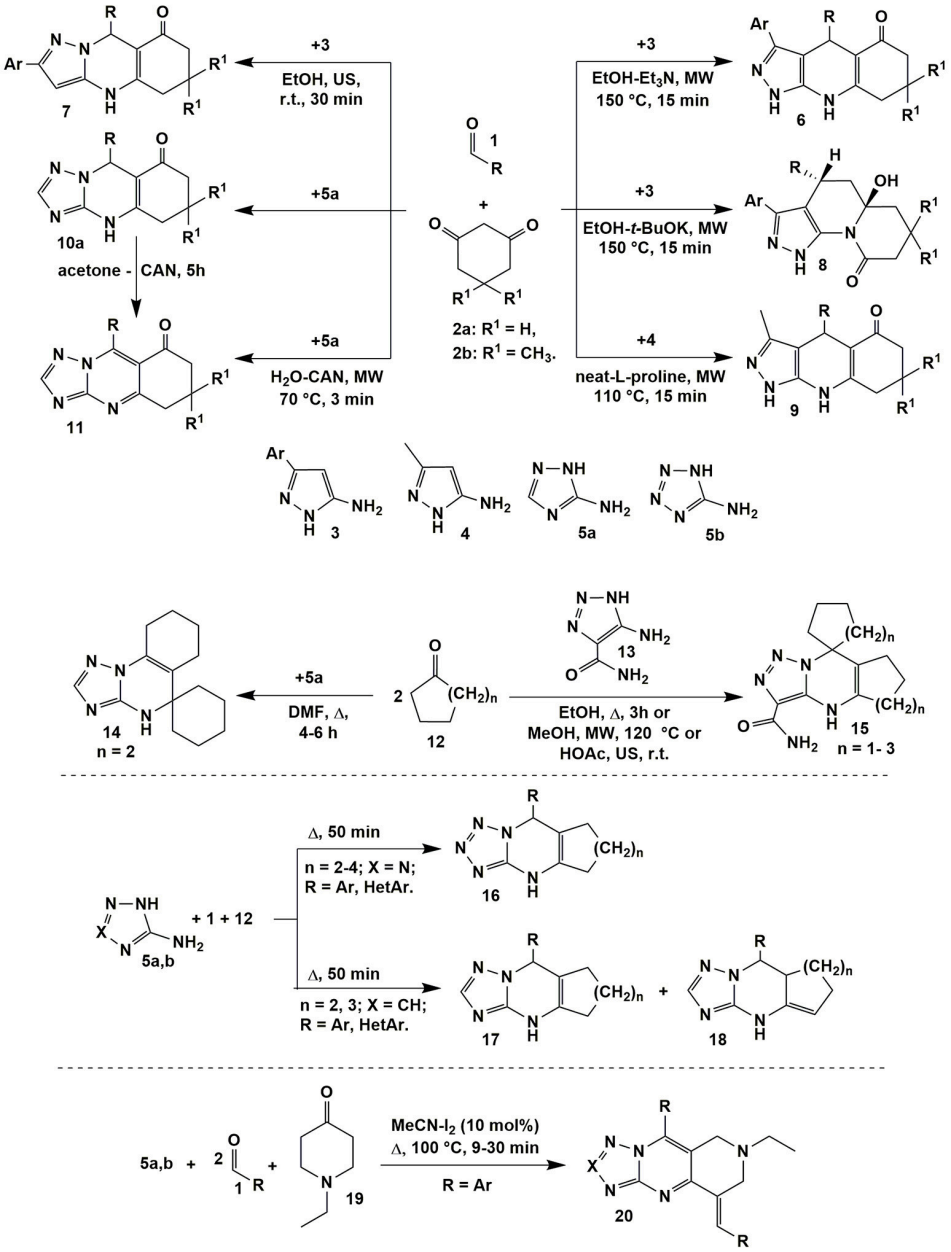
Examples of the condensations involving α-aminoazoles, aldehydes and cyclic carbonyl compounds.

Varying temperature and catalyst allowed authors (Chebanov et al., 2007b, 2008b) to switch the heterocyclization of aromatic aldehydes **1**, 1,3-cyclohexanedione (**2a**) or dimedone (**2b**) with 5-amino-3-arylpyrazoles **3** between two directions with the formation of pyrazoloquinolinenones **6** (EtOH-Et_3_N, MW, 150°C, 15 min) and pyrazoloquinazolinones **7** (EtOH, US, r.t., 30 min) being the products of thermodynamically and kinetically controlled reactions, respectively. Non-classical activation methods led to the reduction in time; moreover, applying microwave activation allowed to carry out the transformations at higher temperatures in comparison with standard heating, thus, additionally favoring reaction regioselectivity in case of thermodynamically controlled pathway (Figure 2).

In the process of optimization, the new multicomponent reaction was found: *t*-BuOK being a stronger nucleophile then Et_3_N attacked the carbonyl group of cyclic 1,3-diketone moiety in the intermediate which resulted in the ring opening and recyclization with the formation of quinolizinones **8**. Later on the greener methodology of obtaining pyrazoloquinolinenones **6** was elaborated using microwave synthesis in water (170°C, 10 min; Andriushchenko et al., 2011). Similar to compounds **6** pyrazoloquinolinenones **9** were synthesized even without solvent using L-proline as a catalyst (MW, 110°C, 15 min; Bhattacharjee et al., 2016).

The analogous to heterocycles **7** linear quinazolinones **10** were obtained on the basis of 3-amino-1,2,4-triazole (**5a**) applying the great variety of conditions (only the endocyclic aminogroup in the position 2 took part in the condensation; Puligoundla et al., 2013; Petrov and Kasatochkin, 2014; Sompalle et al., 2016; Vibhute et al., 2017a,b). It should be noted, that in all cases tetrahydroderivatives **6**–**10** were formed. However, Petrov and Kasatochkin (2014) oxidized partially hydrogenated pyrimidine ring of **10** to obtain compounds **11** using ceric ammonium nitrate (CAN) in acetone. Later on the compounds **11** were synthesized in the three-component reaction of **1**, **2a** and **5a** in water under microwave irradiation also with application of CAN (Figure 2; Sompalle and Roopan, 2016).

Linear tetrahydroquinazolinones of type **10** had been also formed in condensations involving 5-amino-4-aryl-1,2,3-triazole and 5-amino*-N*-aryl-1,2,3-triazole-4-carboxamide (Gladkov et al., 2010), 5-aminotetrazole (Shen et al., 2013; Gein et al., 2015; Kour et al., 2017), 2-aminobenzimidazole (Puligoundla et al., 2013; Maleki et al., 2015), 2-aminoindazole moiety (Palaniraja and Roopan, 2015; Shinde and Jeong, 2016), methyl 5-amino-pyrazole-4-carboxylate (Lipson et al., 2010) and 5-amino-pyrazole-4-carbonitrile (Lipson et al., 2010), 4-aryl-5-aminopyrazole (Petrov and Kasatochkin, 2014). It should be noted, that *N*-unsubstituted 5-amino-1,2,3-triazole-4-carboxamide showed the same behavior and the products of reaction involving carboxamide aminogroup were not separated (Gladkov et al., 2012, 2013).

One of the first angular-structured heterocycles **14** was formed in the ABB′ type multicomponent reaction of 3-amino-1,2,4-triazole (**5a**) (X = CH) and two equivalents of cyclohexanone **12** (*n* = 2) and described by Desenko et al. (1990; Figure 2). When 5-amino-1,2,3-triazole-4-carboxamide (**13**) was introduced into the same condensation linear compounds **15** with other positional orientation of ketone moieties were obtained. The same heterocycles were synthesized in the reaction with cyclopentanone **12** (*n* = 1; Gladkov et al., 2012). However, cycloheptanone (**12**) (*n* = 3) did not react in a multicomponent procedure, therefore, corresponding spiroheterocycles of type **15** were got by the stepwise protocol through the synthesis of cycloheptalidenecycloheptanone [using two equivalents of ketone **12** (*n* = 3)] and further cyclization with 5-amino-1,2,3-triazole-4-carboxamide (**13**). It's worth noting that in case of other ketones **12** (*n* = 0, 1, 2) compounds of type **15** were formed both by the stepwise and by the multicomponent protocols (Figure 2; Gladkov et al., 2012).

ABC type multicomponent cyclization of 5-aminotetrazole (**5b**) (X = N), different aromatic and heteroaromatic aldehydes **1** and ketones **12** (*n* = 2–4) under heating without solvent afforded only one linear isomer **16** (Matveeva et al., 2013), while the same reaction involving 3-amino-1,2,4-triazole (**5a**) (X = CH) resulted in formation of the mixture of isomeric cycloalkatriazolopyrimidines **17** and **18** (Figure 2; Matveeva et al., 2015). The analogous to compounds **16** linear tetrahydrobenzo[*h*]tetrazoloquinazolines were yielded in the condensation of the reagents **1**, **5b** with α-tetralone acting as CH-acid (Kantin and Krasavin, 2016).

When 1-ethyl-4-piperidinone (**19**) was used as a CH-acid in the condensation with two equivalents of aromatic aldehyde **1** and 3-amino-1,2,4-triazole (**5a**) or 5-aminotetrazole (**5b**) or (2-aminobenzimidazole) upon heating (MeCN-I_2_, Δ, 100°C) 1,2,3,4-tetrahydro-pyrido[4,3-*d*]tetrazolo[1,5-*a*]pyrimidines **20** bearing *in situ* oxidized triazolopyrimidine system were formed (Figure 2; Farghaly et al., 2015).

**Figure 3:**
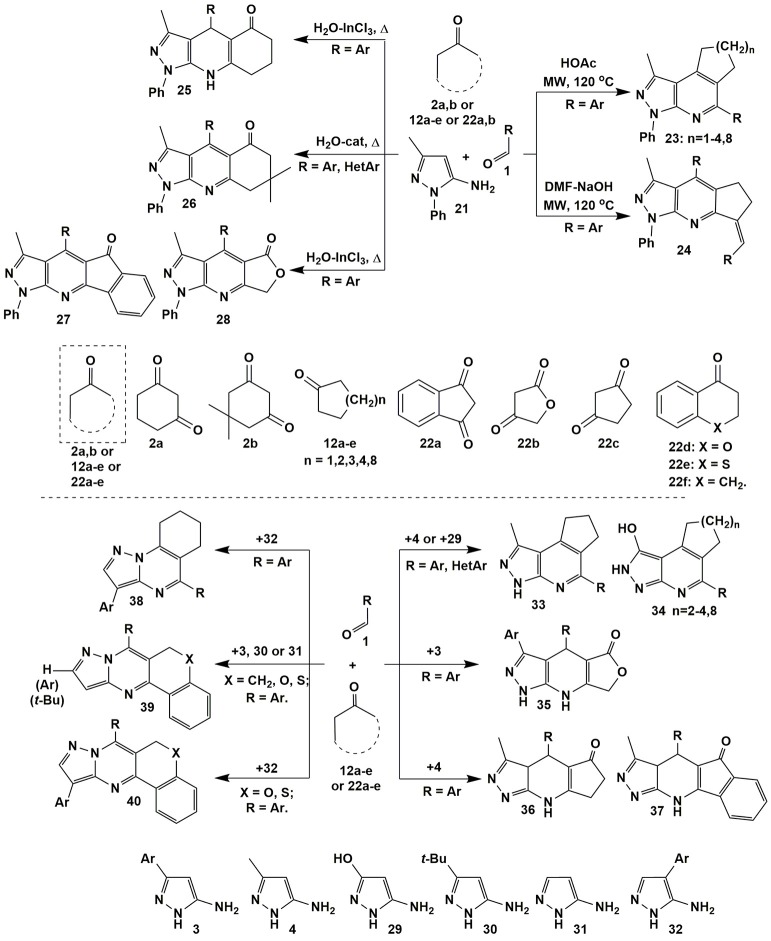
Examples of heterocyclization reactions involving 5-aminopyrazoles, aldehydes and cyclic active methylene compounds.

The condensations involving 5-amino-3-methyl-1-phenylpyrazole (**21**) afforded fused heteroaromatic azolopyridines. Thus, the variation of acid-base properties of the reaction medium led to the change in a sequence of elementary stages in multicomponent reaction involving 5-amino-3-methyl-1-phenylpyrazole (**21**), cyclopentanone (**12a**) and aromatic aldehydes **1** that allowed to switch the reaction between two alternative directions and selectively got positional isomers–angular pyrazolopyridines **23** (*n* = 1; Wang et al., 2011) and linear heterocycles **24**. Another authors (Jiang et al., 2011; Chen et al., 2015) described fused pyrazolopyridines **23** with *n* = 2–4, 8 (Figure 3).

Several publications deal with condensations of the reagents **1** and **21** with 1,3-diketones [dimedone (**2b**) (Karnakar et al., 2012; Wang and Shi, 2012), indane-1,3-dione (**22a**) (Quiroga et al., 2008; Shi et al., 2009) and furane-2,4-dione (**22b**) (Shi et al., 2009)] resulting in the formation of heteroaromatic derivatives **26**–**28**. It's interesting that under the same conditions (H_2_O-InCl_3_, Δ) Khurana et al. (2012) obtained dihydropyrazolopyridines **25** only from 1,3-cyclohexanedione (**2a**), whereas in case of indane-1,3-dione (**22a**) and furane-2,4-dione (**22b**) heteroaromatic compounds **27**, **28** were formed (Figure 3).

Similar to heterocycles **23** angular products **33** (DMF-MeOH, Δ; Lipson et al., 2015) and **34** (HOAc-TFA, MW, 140°C; Jiang et al., 2011) were also got in the condensation with 5-amino-3-methylpyrazole (**4**) and 5-amino-3-hydroxypyrazole (**29**), while the transformations involving 5-amino-4-arylpyrazoles **32** afforded pyrazolopyrimidines **38** (HOAc, Δ; Figure 3; Petrov and Kasatochkin, 2013).

An exhaustive review on the properties of 5-aminopyrazoles as precursors in design and synthesis of fused pyrazoloazines being published yet (Aggarwal and Kumar, 2018) describes the reaction of 5-amino-3-methyl-1-phenylpyrazole (**21**) and aromatic aldehydes **1** with 4-hydroxycoumarin, where 3 types of possible products (4,7-dihydropyrazolo[3,4-*b*]pyridine-, aromatized pyrazolo[3,4-*b*]pyridine derivatives and the product of C–O bond cleavage from cyclic ester) were formed depending on the type of solvent and temperature. MCRs of 1-aryl-3-indolyl-5-aminopyrazoles, cyclic β-diketones (dimedone, cyclopentanedione, indane-1,3-dione) and aromatic aldehydes also gave dihydro- and aromatized pyrazolo[3,4-*b*]pyridine derivatives, what is more, dihydropyrazolo[3,4-*b*]pyridines formed could be transformed into their heteroaromatized analogs by prolonged heating in acetonitrile with DDQ (2,3-dichloro-5,6-dicyanobenzoquinone).

It should be noted, that reactions with chroman-4-one (**22d**), thiochroman-4-one (**22e**) or 3,4-dihydronaphthalen-1(2*H*)-one (**22f**) (EtOH-*t-*BuOK, Δ) despite of the origin or position of the substituent in pyrazole afforded only heteroaromatized “classical” Biginelly-type pyrazolopyrimidines **39** and **40** (Saikia et al., 2014). Condensations of 1,3-diketones with 3-substituted 5-aminopyrazoles **3** and **4** also led exclusively to linear dihydropyrazolopyridinones **35** (Hatti et al., 2015) and **36**, **37** (Lipson et al., 2015), correspondingly (Figure 3).

**Figure 4:**
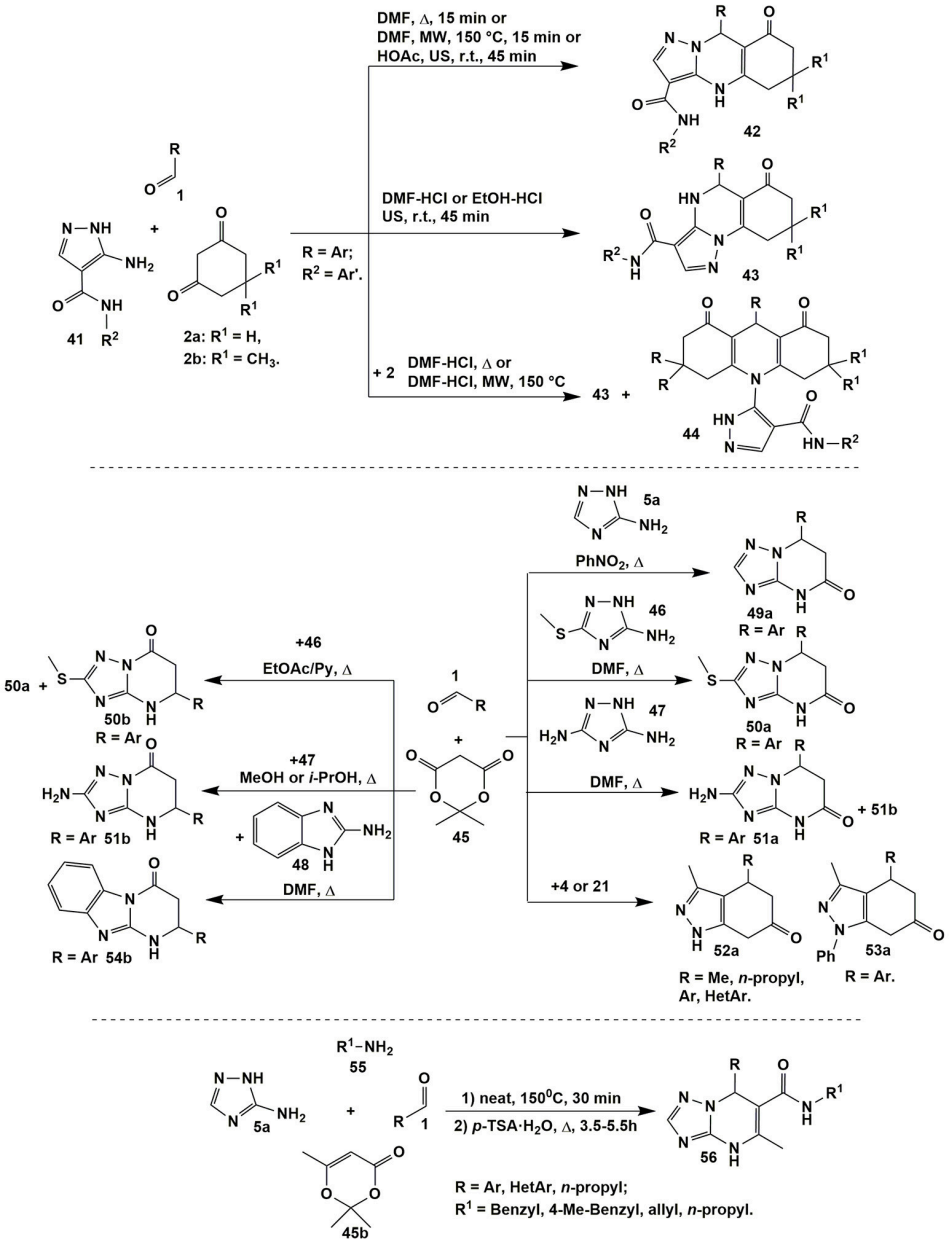
Examples of azoloazines synthesis via reactions involving 5-aminopyrazole-4-carboxamides of 3-amino-1,2,4-triazoles derivatives.

Using 5-aminopyrazoles **41** bearing carboxamide fragment in the fourth position in condensations with 1,3-cyclohexanediones **2a,b** and aromatic aldehydes **1** led to widening the scope of target compounds whereas varying reaction parameters and applying non-classical methods of activation (ultrasonication and microwave irradiation) allowed to switch cyclizations between several directions (Figure 4; Chebanov et al., 2012b).

Thus, condensation of starting reagents **1**, **2a,b**, and **41** upon heating or MW irradiating in DMF or ultrasonication in HOAc at room temperature always afforded tricyclic dihydropyrimidines **42**. Addition of catalytic amounts of hydrochloric acid resulted in switching the reaction to another direction and yielding positionally isomeric angular compounds **43**. Implementation of the third route with the formation of acrydindiones **44** occurred upon increasing the temperature and introducing the double excess of diketone **2** (Chebanov et al., 2012b).

Meldrum's acid is also widely used as a building block for the synthesis of azoloazine systems. A significant contribution to the study of the condensations involving aminoazoles and aldehydes with Meldrum's acid was made by Lipson's group (Figure 4; Lipson and Gorobets, 2009). It was established, that in some cases these multicomponent reactions afforded positional isomers. For example, condensations involving 3-amino-5-methylthio-1,2,4-triazole (**46**) gave 5-pyrimidinones **50a** or 7-pyrimidinones **50b** with impurities of **50a** depending on solvent and catalyst (Lipson and Gorobets, 2009).

The opposite situation was observed in case of 3,5-diamino-1,2,4-triazole (**47**): 5-pyrimidinones **51a** were obtained only in mixture with isomers **51b**. At the same time the latter compounds **51b** were isolated in a pure state after changing DMF to methanol or isopropanol (Lipson and Gorobets, 2009). When 3-amino-1,2,4-triazole **5a** (Lipson and Gorobets, 2009) took part in the condensation with compounds **1** and **45** only 5-pyrimidinones **49a** were isolated while the reaction involving 2-aminobenzimidazole **48** (Sheibani et al., 2012) afforded only 7-pyrimidinones **54b** (Figure 4).

Condensations of 5-amino-3-methylpyrazole (**4**) (Lipson and Gorobets, 2009; Zhong et al., 2013) or 5-amino-3-methyl-*N*-phenylpyrazole (**21**) (Shi et al., 2011) with aldehydes **1** or arylglyoxals (Petrova et al., 2014) and Meldrum's acid **45** under various conditions gave 5-pyrimidinones **52a** and **53a**, correspondingly (Figure 4); similar heterocycles were obtained from 2-aminobenzothiazole (Arya et al., 2012).

An interesting one-pot four-component reaction was described by Shaabani et al. (2015): [1,2,4]triazolo[1,5-*a*]pyrimidine-6-carboxamide derivatives **56** were synthesized via reaction of primary aliphatic or aromatic amines **55** and 2,2,6-trimethyl-4*H*-1,3-dioxin-4-one (**45b**) (heating under solvent-free conditions, 150°C, 30 min) followed by the subsequent condensation with 3-amino-1,2,4-triazole (**5a**) and aliphatic or aromatic aldehydes **1** (H_2_O-*p*-TSA, Δ, 3.5–4.5 h; Figure 4).

**Figure 5:**
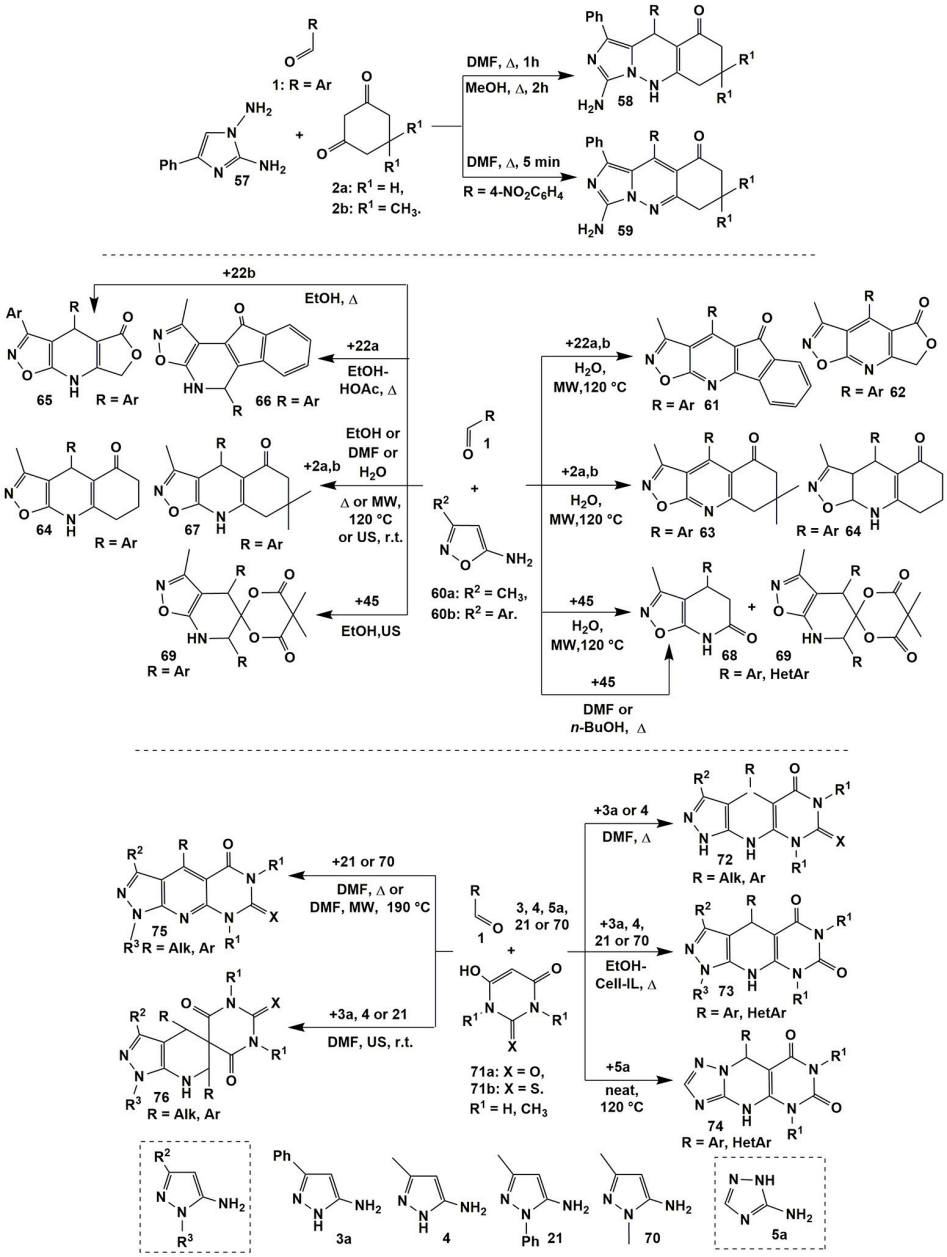
Diversity of compounds generated by using the conditions based divergence strategy.

The presence of four non-equivalent reaction centers in 1,2-diamino-4-phenylimidazole (**57**) makes possible new alternative reaction routes with electrophilic reagents. Due to the lower nucleophilicity of exocyclic amino groups in comparison with endocyclic CH-group, 1,2-diaminoimidazoles in the reactions with α,β-unsaturated ketones, their mono- and dibromo derivatives, with aroylacrylic acids, and in the three-component reactions with aldehydes and Meldrum's acid formed not triazepine fragments but pyridazine and pyrimidine systems fused with azole cycle (Lipson et al., 2012). This fact was also confirmed in the multicomponent reaction involving 1,3-cyclohexanediones **2a,b** upon boiling in DMF (1 h) or methanol (2 h) which resulted in the formation of dihydroimidazo[1,5-*b*]cinnolinones **58**. Only in case of 4-nitrobenzaldehyde **1** the short-term boiling the compounds **1**, **2** and **57** in DMF led to the formation of heteroaromatic derivatives **59** (Figure 5; Lipson et al., 2012).

*N-*Unsubstituted 2-aminoimidazole exhibited similar properties (lower nucleophilicity of exocyclic aminogroup than endocyclic reaction centers) in the reactions with aromatic aldehydes and different CH-acids (dimedone, barbituric acid), but instead of cyclic products the treatments yielded Michael adducts (with participation of CH-center in the position 3; Andriushchenko et al., 2013). On the other hand, the formation of Mannich bases in the similar reactions involving 2-aminothiazole indicates on the higher reactivity of its exocyclic NH_2_-group in comparison with the endocyclic nucleophilic centers (Ghatole et al., 2015).

There is contradictory data in the literature about three-component reactions involving 5-amino-3-methyl(aryl)isoxazole **60** and aromatic aldehydes **1** with various 1,3-diketones. Thus, Tu et al. (2009) carried out that condensations under microwave irradiation at 120°C in water and synthetized isoxazolo[5,4-*b*]pyridines **61–63**. Surprisingly, heterocyclization involving 1,3-cyclohexanedione (**2a**) under the same conditions led to dihydroderivatives **64** (Figure 5).

Later on Muravyova et al. (2013) carried out wide screening of the reaction conditions and found that in all cases including those ones described in the work of Tu et al. (2009) [in the model reactions with 1,3-cyclohexanedione (**2a**) and dimedone (**2b**)] dihydroisoxazole[5,4-*b*]pyridines **64** and **67**, respectively, had been formed. Annulated with furane-2,4-dione moiety dihydroisoxazolo[5,4-*b*]pyridines **65** were yielded upon boiling the starting materials in EtOH (Kamal et al., 2011). Hamama et al. (2012) managed to synthesize angular heterocycles **66** on the basis of indane-1,3-dione (**22a**) [EtOH-HOAc (15:1), Δ], however, we suggest that there is not enough data proving the structure of compounds **66** (Figure 5).

When Meldrum's acid was used, different compounds−4,7-dihydroisoxazolo[5,4-*b*]pyridine-6(5*H*)-ones **68** (6–9 min) or spiroheterocycles **69** (9–13 min) were got under almost the same conditions by Tu et al. in two consecutive publications (Tu et al., 2009; Ma et al., 2010). Later on Morozova et al. (2017) reproduced the synthesis of compounds **68** and **69** under the same conditions, however, all the attempts gave only the mixtures of compounds **68** and **69** or heterocycles **69** were isolated in the lower yields than in the previous work (Tu et al., 2009). Therefore, Morozova et al. (2017) studied in details the reactions of 5-amino-3-methylisoxazole (**60a**) and aromatic aldehydes **1** with Meldrum's acid (**45**) and developed the preparative methodologies for selective synthesis of the products **68** (boiling in DMF or *n*-BuOH) and **69** (ultrasonication in EtOH).

The detailed study of the reactions of 5-aminopyrazoles (**3a**, **4**, **21**, and **70**) with aromatic aldehydes **1** and barbituric acids **71** (a: X = O, b: X = S) showed that varying temperature and type of R^3^-substituent in 5-aminopyrazole were the main factors of switching the direction to produce different final compounds (Muravyova et al., 2009). Thus, in case of pyrazoles **3a** and **4** (R^3^ = H) boiling the reagents in DMF led to dihydropyrazolopyridopyrimidines **72** (Muravyova et al., 2009) whereas pyrazoles **70** and **21** bearing methyl or aryl R^3^-substituents (both of electron donor and acceptor origin) afforded heteroaromatized derivatives **75** (Muravyova et al., 2009). It's interesting to note that Satasia et al. (2014) got dihydropyrazolopyridopyrimidines **73** in case of all the pyrazoles (R^3^ = H, Ph) under refluxing the starting materials in ethanol with adding cellulose supported ionic liquid (Cell-IL) (Figure 5).

Similar to heterocycles **75** other heteroaromatic compounds were synthesized earlier by the group of Shi (Shi et al., 2008) in the reaction of 5-amino-3-methyl-1-phenylpyrazole (**21**), aromatic aldehydes **1** and barbituric acids **71a** (R^1^ = H, CH_3_; H_2_O-*p*-TSA, MW, 140°C) whereas the condensation involving thiobarbituric acids **71b** (R^1^ = H; neat-*p*-TSA, MW, 100°C) afforded corresponding dihydropyrazolopyridopyrimidines of type **73** (El-Emary and El-Mohsen, 2012). Later on dihydropyridopyrimidines **74** were synthesized starting from 3-amino-1,2,4-triazole (**5a**) (Karami et al., 2015a) and 2-aminobenzimidazole (Kaur G. et al., 2015).

Ultrasonication at room temperature of compounds **1** and **71** with 5-aminopyrazoles **3a**, **4** or **21** afforded new spiroheterocyclic systems **76** (Muravyova et al., 2009) that had not been formed in the previously described reactions with 1,3-diketones. Analogous reactions involving 5-amino-3-methylisoxazole were studied in several works. After wide screening the reaction conditions similar to compounds **76** spiroheterocycles were the only product obtained in the condensation of 5-amino-3-methylisoxazole (**60a**) with aromatic aldehydes **1** and barbituric acids **71** under MW irradiation in water (9–13 min; Jiang et al., 2012) or by ultrasonication in ethanol (r.t., 2 h; Morozova et al., 2017).

Replacing 5-amino-3-methylisoxazole (**60a**) with isomeric 3-amino-5-methylisoxazole did not contribute to the formation of new heterocyclic fragments: it didn't react with aldehydes and barbituric acids or Meldrum's acid (arylidene derivatives were yielded); heating 3-amino-5-methylisoxazole with aldehydes and Meldrum's acid in DMF or ethanol afforded only its acylated derivative (Morozova et al., 2017). Such a reactivity of 3-amino-5-methylisoxazole is consistent with other literature data, which describes its chemical behavior in the synthesis of pyrrolones (Ryabukhin et al., 2012), in the Hantzch (Rajanarendar et al., 2011b) and Betti (Shafiee et al., 2012) reactions as well as in four-component condensation with formation of imidazole moiety (Rajanarendar et al., 2011a).

**Figure 6:**
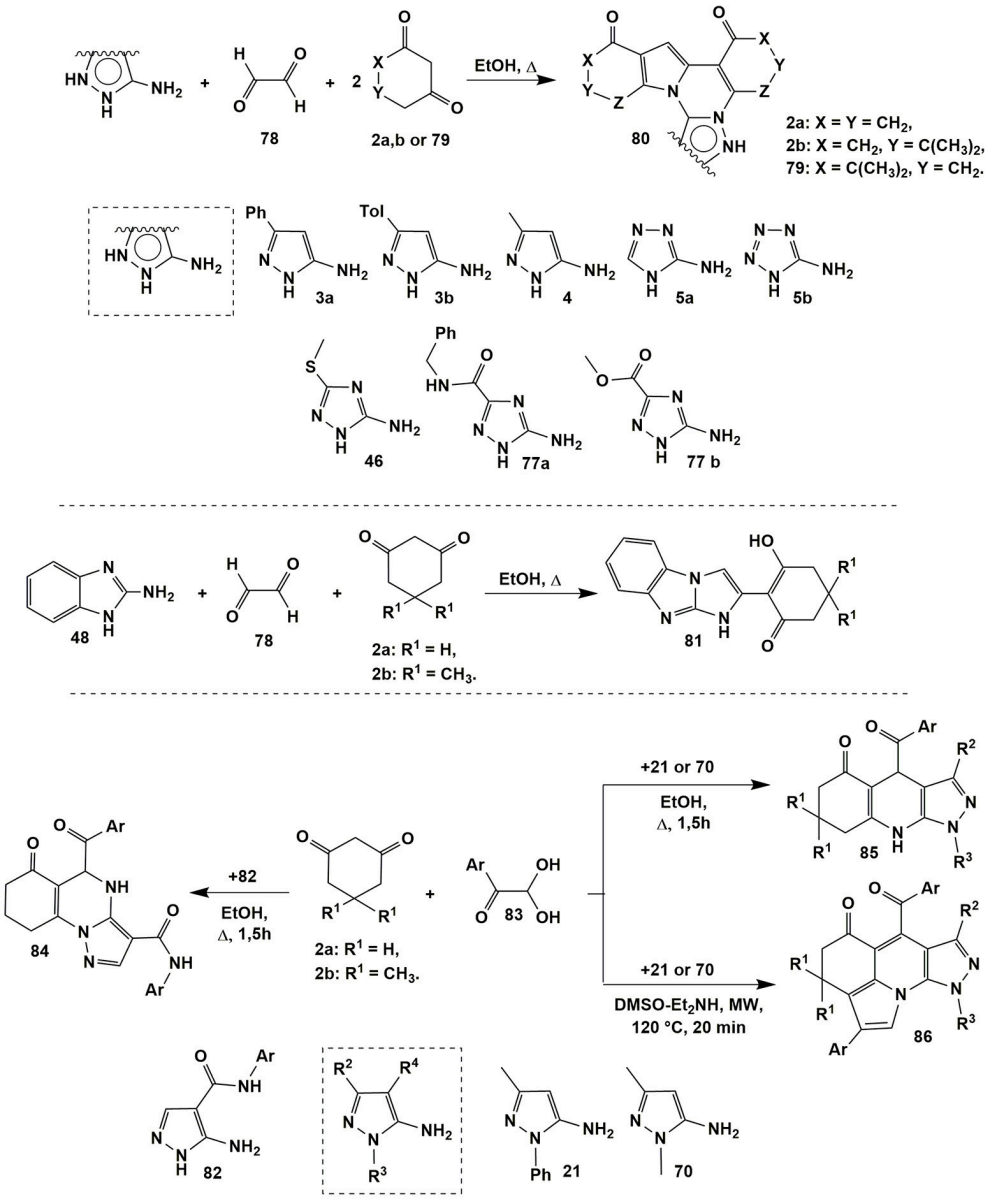
MCR*s* involving glyoxales derivatives acting as carbonyl compounds.

When glyoxales and arylglyoxales were used instead of aldehydes in multicomponent reactions, some additional reaction pathways could be implemented. Thus, Petrova et al. (2013b) studied the condensations of wide spectrum of aminoazoles (**3a**, **3b**, **4**, **5a**, **5b**, **46**, **77a**, and **77b**) with glyoxales **78** and 1,3-diketones **2a,b** or **79** upon refluxing in ethanol (for 30–40 min in case of 5-aminopyrazoles; 2.5–3 h in case of 1,2,4-triazoles and for 10 h in case of 5-aminotetrazole) and obtained the novel heterocyclic system–indolo[1,2-*c*]polycyclic compounds **80** (Figure 6) instead of the expected 4,5,6,7,8,9-hexahydro-8-oxoazolo[5,1-*b*]quinazoline-9-carbaldehyde derivatives (similar to compounds **7** or **10**, see Figure 2).

In case of 2-aminobenzimidazole **48** its condensation with glyoxal **78** and 1,3-diketones **2a,b** under the same conditions led to the formation of benzo[*d*]imidazo[1,2*-a*]benzimidazoles **81** containing only one cyclohexanedione fragment in their structure (Figure 6; Petrova et al., 2013b).

When arylglyoxals **83** had been introduced into condensation with 5-aminopyrazoles **82** having arylcarboxamide group in the fourth position, angular pyrazoloquinazolinones **84** (similar to compounds **43**, Figure 4) were formed under refluxing in EtOH (Figure 6; Petrova et al., 2013a). Other 1- and 3-substituted 5-aminopyrazoles **21** and **70** (R^4^ = H) in the condensation with compounds **2a,b** and **83** under the same conditions gave expected pyrazoloquinolinenones **85**. Applying microwave irradiation in DMSO-Et_2_NH (120°C, 20 min) to the mixture of diketones **2a,b**, pyrazoles **21** or **70** and two equivalents of arylglyoxals **83** afforded an elegant four-component domino reaction leading to polycyclic compounds **86** (Wang et al., 2015b; Figure 6).

**Figure 7:**
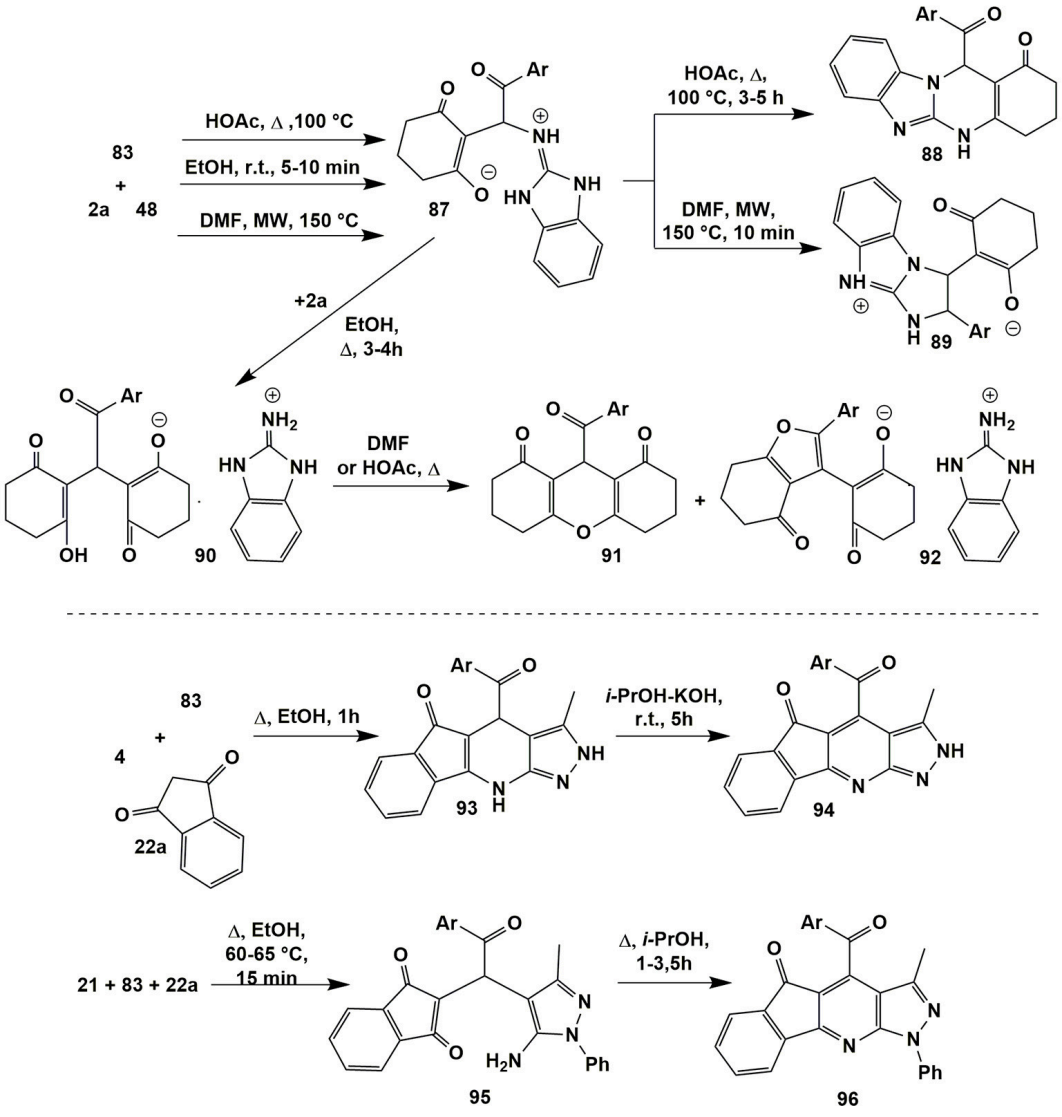
Condensations of α-aminoazoles involving arylglyoxales and cyclic CH-acids.

A multicomponent reaction involving 2-aminobenzimidazole (**48**), cyclohexanedione (**2a**) and arylglyoxales **83** was thoroughly studied by Petrova et al. (2015b); all the compounds including intermediates were isolated in individual form, characterized and their structures were proven with the help of X-Ray analysis (Figure 7).

It was established that the reaction of the reagents **2a**, **48**, and **83** in ethanol at room temperature for 5–10 min yielded Michael adduct **87** that remained stable upon latter refluxing in primary alcohols. However, after adding the second equivalent of cyclohexanedione (**2a**) and further prolonged refluxing the reaction mixture in ethanol salts **90** were isolated. All the attempts to convert them into the products of condensation with 2-aminobenzimidazole (**48**) upon refluxing in DMF or acetic acid or fusion under neat conditions were unsuccessful. In all cases the mixtures of xanthenediones **91** and salts **92** were obtained.

The authors (Petrova et al., 2015b) established that Michael adduct **87** had been also formed after heating the starting reagents in acetic acid at 100°C; after longer heating, they turned into condensed quinazolinones **88**. Microwave irradiation of the starting reagents **2a**, **48**, and **83** in DMF (150°C) afforded to get heterocycles **89** as the main products (Figure 7).

Linear pyrazolopyridinones **93** (Petrova et al., 2015a) were synthesized by the condensation involving 5-amino-3-methyl-pyrazole (**4**) and arylglyoxales **83** with indane-1,3-dione (**22a**) upon short-term heating in ethanol. Further prolonged treatment of compounds **93** at room temperature in isopropanol with the addition of KOH led to their transformation into heteroaromatic derivatives **94**. When the authors (Petrova et al., 2015a) applied 5-aminopyrazole **21** containing an aryl substituent in the first position, in the reaction with compounds **83** and **22a** in ethanol (60–65°C, 15 min) the Michael adduct **95** was initially isolated. Further prolonged boiling the compound **95** in isopropanol led directly to heteroaromatic derivatives **96** whereas dihydroindeno[1,2-*b*]pyrazolo[4,3-*e*]pyridinones of type **93** failed to be found (Figure 7).

### Multicomponent reactions of aminoazoles involving non-cyclic CH-acids

Multicomponent reactions of aminoazoles and carbonyl compounds often involve such CH-acids as enolizable ketones, 1,3-dicarbonyl compounds (acetoacetic acid and its derivatives, 1,3-diketones, ketosulfones), 1,2-dicarbonyl compounds (pyruvic acid and its derivatives), malonic acid and its derivatives, cyanoacetamide etc.

**Figure 8:**
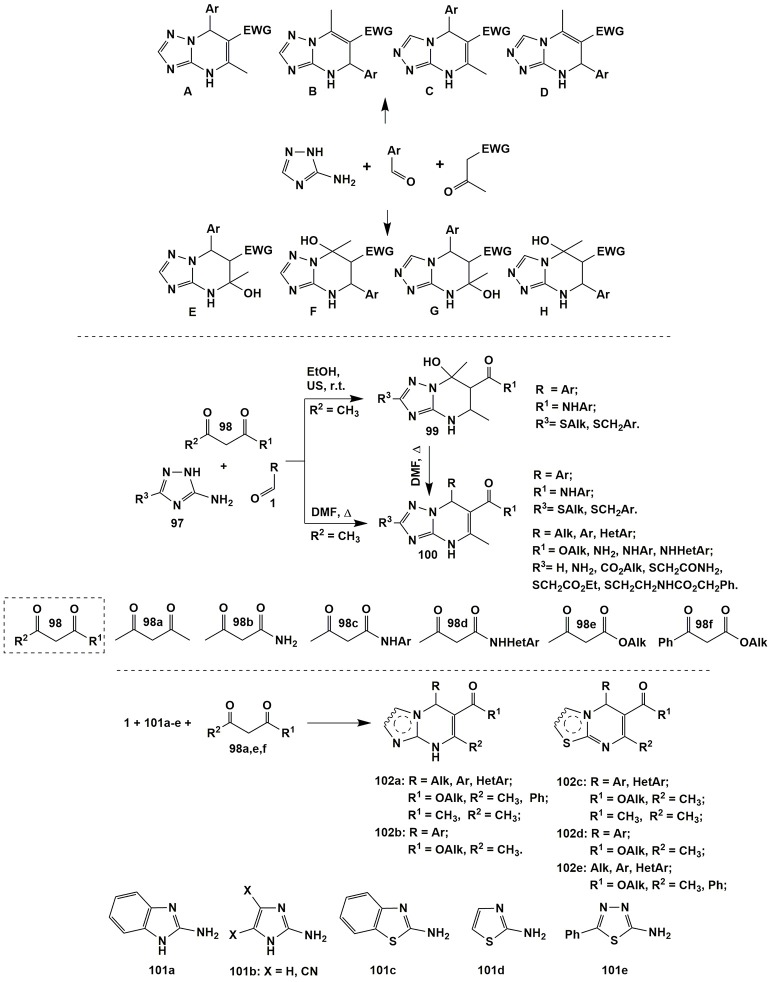
The diversity and complexity of Biginelly-type transformations involving α-aminoazoles.

Recently published review by Sedash et al. (2012) clearly illustrates the diversity and complexity of MCRs of aminoazoles, carbonyl compounds and non-cyclic CH-acids on the example of the Biginelly-type transformations involving 3-amino-1,2,4-triazoles as 1,3-binucleophiles. It was shown that the stepwise character of the MCRs themselves and polyfunctional character of that 1,3-binucleophile could lead to at least eight possible products **A–H** from one set of the starting reagents usually depending on the reaction conditions (and sometimes specific structure of the starting reagents themselves). The pairs **A-B**, **C-D**, **E-F**, and **G-F** could be considered as positional isomers whereas the pairs **A-C**, **B-D**, **E-G**, and **F-H**–as regioisomers. As a consequence of such diversity of the possible reaction products their structural elucidation often becomes problematic (Figure 8).

The authors (Sedash et al., 2012) explored the literature dealing with this type of reactions and concluded that the existing data about the structure of the reaction products **A–H** was not always comprehensive and found that most of the literature sources concerning Biginelli-like MCRs involving 3-amino-1,2,4-triazoles had described structure **A** as the most usual product under quite harsh conditions and different reagents (acetophenone, ethyl and methyl esters of acetoacetic acid and its fluorinated derivatives, substituted amides of acetoacetic acids, pyruvic acid, 1-(methylsulfonyl)propan-2-one and 2-(methylsulfonyl)-1-phenylethanone, aliphatic ketones). The structure **C** was sometimes reported as a side product accompanying the formation of the main product **A** (in the reactions with pyruvic acid or methyl acetoacetate). Only the condensation involving acetylpyrazole derivative afforded compound of structure **C** as the only product (Ali et al., 2016). Tetrahydroderivatives of type **F** were described in the reactions under mild conditions (with phenylpyruvic acid or ethyl acetoacetate). Products of **E**-type could be obtained on the basis of fluorinated esters of acetoacetic acid and further converted into **A**-type heterocycles thus being the products of kinetically and thermodynamically controlled reactions. The products of structure **B** could be formed when two molecules of acetophenone (or cyclohexanone, see compound **14**, Figure 2) reacted with 3-amino-1,2,4-triazole derivatives. The products **D** were not obtained by Biginelli-like MCRs, they could be synthesized using other approaches whereas products of structure **G** and **H** were not described at all (Figure 8; Sedash et al., 2012).

The conclusions of Sedash et al. about preferential formation of compounds with the structure of type **A** in the multicomponent reactions involving 3-amino-1,2,4-triazole were confirmed by the subsequent publications of other authors: 4,7-dihydroazolo[1,5-*a*]pyrimidines were synthesized from 3-amino-1,2,4-triazole or 5-aminotetrazole, aromatic aldehydes and acetone, α-acetyl-butyrolactone, acetylacetone or acetoacetic acid derivatives (Ryabukhin et al., 2011; Gein et al., 2012, 2014; Kumari et al., 2012; Li et al., 2012; Liu et al., 2012b; Rajua et al., 2012; Ghorbani-Vaghei et al., 2013; El Rady, 2014; Haleel et al., 2014; Bhatt et al., 2015; Adrom et al., 2016; Komykhov et al., 2016, 2017; Gümüş et al., 2017b; Kour et al., 2017; Maleki et al., 2017).

An example illustrating the dependence of the reaction direction on the conditions is given in work of Muravyova et al. (2011) Varying temperature and using ultrasonic activation allowed to switch MCR involving acetoacetamides **98b** (R^1^ = NHAr, R^2^ = CH_3_), aromatic aldehydes **1** and substituted 3-amino-1,2,4-triazole **97** (R^3^ = SAlk, SCH_2_Ar) between kinetically- and thermodynamically-controlled directions and selectively obtain tetrahydro- or dihydroderivatives **99** or **100**, correspondingly. Later on Wang et al. (2015a) expanded the list of dihydropyrimidines **100** by using other substituted triazoles **97** (R^3^ = H, NH_2_, CO_2_Alk, SCH_2_CONH_2_, SCH_2_CO_2_C_2_H_5_, SCH_2_CH_2_NHCO_2_CH_2_Ph) and acetoacetic acid derivatives **98** (R^1^ = OAlk, NH_2_, NHAr, NHHetAr, R^2^ = CH_3_; Figure 8).

It is worth noting that the analogous behavior of 5-aminopyrazoles substituted in the fourth position with electron-withdrawing groups [CN, CO_2_CH_3_, CONH_2_ (Muravyova et al., 2011)] in the condensations being similar to the described above and leading to tetrahydro- and dihydropyrimidines of types **99** and **100** as well.

Dihydropyrimidine systems **102a–e** (Figure 8) were also formed in condensation of acetoacetic acid and its derivatives (or acetylacetone) **98** with 2-aminobenzimidazole (**101a**) [under neat conditions-ionic liquid catalyzed, US, 50°C (Reddy et al., 2016) etc. (Ryabukhin et al., 2011; Shaterian et al., 2014; Kaur N. et al., 2015; Abedini et al., 2016; Dam et al., 2016)], 2-aminoimidazoles **101b** [DMF-(CH_3_)_3_SiCl, US, r.t. (Ryabukhin et al., 2011)], 2-aminobenzothiazole (**101c**) (MeOH-HCl, Δ (Chikhale et al., 2015) etc. (Zhao et al., 2013; Atar et al., 2014; Shaterian et al., 2014; Moradi et al., 2015), 2-aminothiazole **101d** [HOAc, MW, 80°C (Zhao et al., 2013) etc. (Batool et al., 2016; Dam et al., 2016; Tan et al., 2016)] and 2-amino-1,3,4-thiadiazole **101e** (HOAc, MW, 65°C; Zhao et al., 2014).

It should be noted, that Thorat et al. (2013) obtained imidazopyrimidines (EtOH-ionic liquid catalyzed, r.t.) with another positional orientation of substituents than in compounds **102b**. However, there was not enough data (2D NMR experiments or X-Ray analysis) proving that structure while the structure of azolopyrimidines **102** was proven with the help of X-Ray analysis in cases of heterocycles **102d** (Zhao et al., 2013) and **102e** (Zhao et al., 2014).

**Figure 9:**
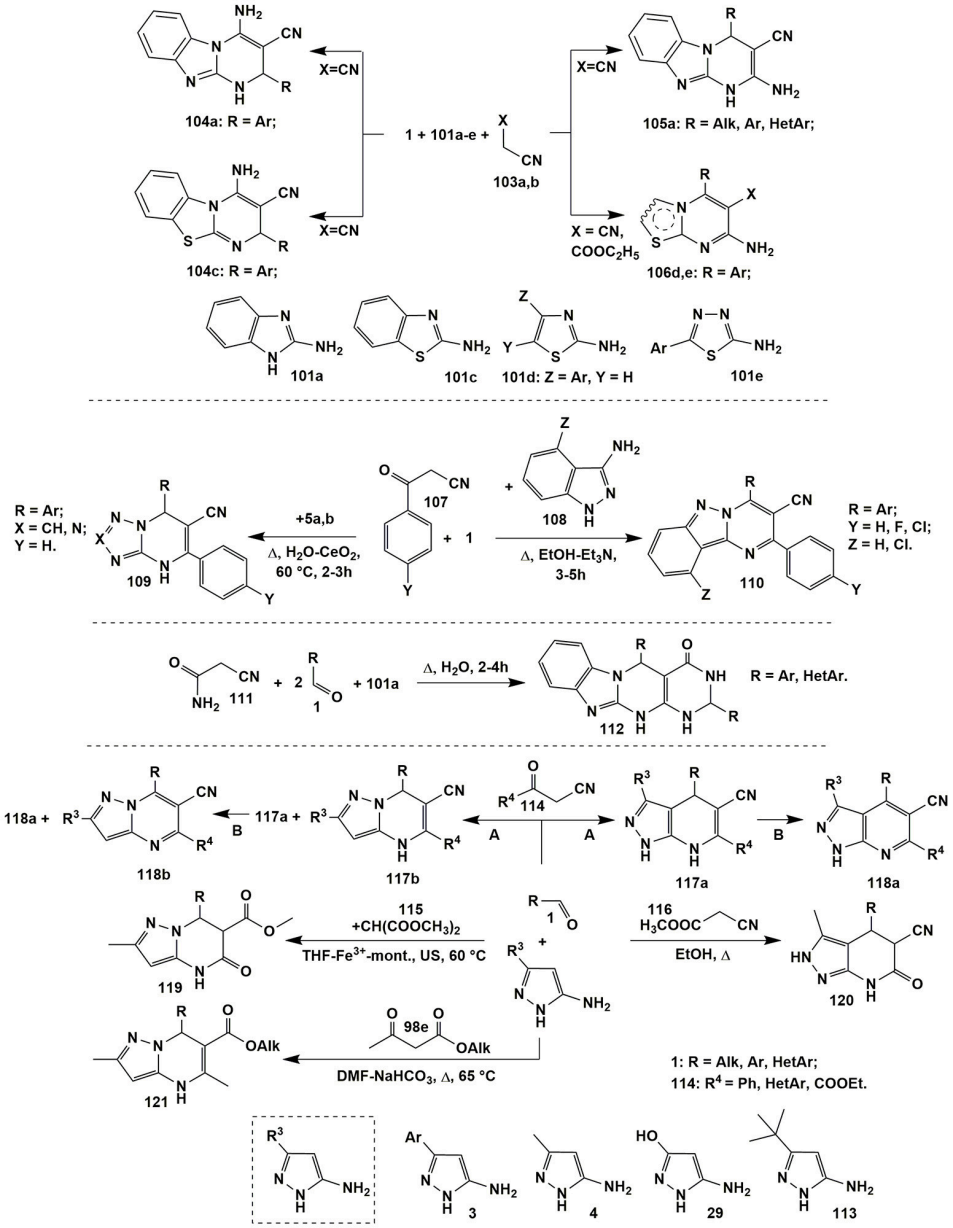
Examples of cyclizations involving α-aminoazoles, aldehydes and non-cyclic carbonyl compounds.

Formation of pyrimidines **105a** and **106d,e** was also observed in the reactions with the CH-acids **103**: malononitrile (**103a**) (X = CN) or ethyl 2-cyanoacetate (**103b**) (X = COOC_2_H_5_). The condensation of aldehydes **1**, malononitrile (**103a**) and 2-aminobenzimidazole (**101a**) under the variety of conditions [neat, poly(vinylpyrrolidonium) perchlorate catalyzed, 100°C (Abedini et al., 2016); neat-*p*-TSA(10%), 80°C (Reddy et al., 2014b); EtOH-Fe_3_O_4_@IM, Δ (Hemmati et al., 2016); PEG-H_2_O (4:1), Δ (Survase et al., 2017)] afforded dihydrobenzo[4,5]imidazo[1,2-*a*]pyrimidines **105a**. When 2-aminothiazoles **101d** and 2-amino-1,3,4-thiadiazoles **101e** reacted with aldehydes **1** and malononitrile (**103a**) or ethyl 2-cyanoacetate (**103b**), [EtOH-H_2_O, MW, 100°C (Sahi and Paul, 2016)] pyrimidines **106d,e** were isolated (Figure 9).

On the other hand, a lot of earlier publications stated that the MCR of malononitrile (**103a**) or ethyl 2-cyanoacetate (**103b**) and aromatic aldehydes **1** with 2-aminobenzimidazole (**101a**) or 2-aminobenzothiazole (**101c**) afforded the products **104a** (EtOH-alum, 70°C (Karimi and Bayat, 2011); H_2_O, Δ (Risley et al., 2014); neat with alkyl disulfamic acid functionalized magnetic nanoparticles, 90°C (Bodaghifard et al., 2016) and **104c** [MeCN-MgO, Δ (Sheibani and Babaie, 2013)] with the other positional orientation than in heterocycles **105a** and **106d,e**. Since a lot of authors provided no sufficient experiment to prove the stated structures the data described above was contradictory. For example, some publications operate with 2D NMR studies: Hemmati et al. (2016) observed NOE between the signals of pyrimidine CH and aromatic benzimidazole protons and indicated on the structure **105a**, whereas Karimi and Bayat (2011) didn't observe such NOE correlation and suggested the formation of isomeric structure **104a**. Some authors on the basis of references for similar compounds having the results of the X-Ray analysis indicated on the formation of isomer **104a** as well.

Similar to compounds **106** heteroaromatized pyrazolopyridines were obtained in the condensations of malononitrile (**103a**) and arylglyoxals **83** both with 5-amino-3-methyl(aryl)-1-phenylpyrazole and *N*-unsubstituted 5-amino-3-methylpyrazole (Petrova et al., 2016).

A facile and efficient cascade reaction of 3-oxo-3-arylpropanenitrile **107** and aromatic aldehydes **1** with substituted 1*H*-indazol-3-amines **108** upon refluxing in EtOH-Et_3_N medium (3–5 h) under metal-free conditions afforded pyrimido[1,2-*b*]indazole-3-carbonitrile derivatives **110** (Li et al., 2017) while their dihydro analogs **109** (Suresh et al., 2016) were synthesized in the condensation of the starting reagents **1** and **107** with 3-amino-1,2,4-triazole (**5a**) (X = CH) or 5-aminotetrazole (**5b**) (X = N) under heating in water with adding of nano CeO_2_ catalyst (60°C, 2–3 h). Tetracyclic derivatives **112** were formed in the reaction involving 2-cyanoacetamide (**111**) with 2-aminobenzimidazole (**101a**) and aldehydes **1** followed by the further cyclization with the second equivalent of aldehyde **1** by heating in water for 2–4 h (Liu et al., 2012a) or at room temperature in polyethylene glycol for 1 h (Figure 9; Reddy et al., 2014a).

The introduction of 1- and 4-unsubstituted 5-aminopyrazoles (**3**, **4**, **29** or **113**) to the reactions with aldehydes and CH-acids enables the formation of the regioisomers. Thus, 4,7-dihydropyrazolopyridines **117a** were the products of condensation of 5-aminopyrazoles **3, 4** or **29** and aldehydes **1** with substituted 3-oxopropanenitriles **114** (A: DMF-Et_3_N, Δ). The volatile substances were removed from the reaction mixture and the residue was oxidized with sodium nitrite in acetic acid (B), which resulted in isolation of pyrazolopyridines **118a** (Hill, 2016). Regioisomeric pyrazolopyrimidines **117b** were formed under the same conditions when 5-aminopyrazoles contained a sufficiently large substituent R^3^ at position 3 (for example, *tert*-butyl in compound **113**) which complicated the electrophilic aromatic substitution with the participation of the C4 nucleophilic center in the aminopyrazole **113** and led to cyclization into compounds **117b**. The authors (Hill, 2016) also discovered the steric influence of an aldehyde component **1** on the ratio of products **118a** and **118b** in the mixture (Figure 9). Analogous to heterocycles **117a** dihydropyrazolopyridines were isolated as a result of heating the compounds **1**, **4** and **114** in ethanol with adding Fe(III)-montmorillonite (Mamaghani et al., 2013).

When methyl cyanoacetate **116** was introduced into the reaction with compounds **1** and **4** under refluxing in ethanol (Mahdavinia and Rahmati, 2015) or ethanol with *p*-TSA (Rahmati, 2010) 6-oxo-4,5,6,7-tetrahydro-2*H*-pyrazolo[3,4-*b*]pyridines **120** were obtained. Isomeric pyrazolopyrimidinones **119** (Hossein Nia et al., 2014) were isolated in the condensation involving dimethyl malonate **115** under ultrasonication in THF with adding of Fe^3+^-montmorillonite (Figure 9). Cyclizations involving acetoacetic acid derivative **98e** proceeded involving endocyclic amino group of 5-aminopyrazole **4** with the formation of dihydropyrimidines **121** (Finlay et al., 2012, 2013).

**Figure 10:**
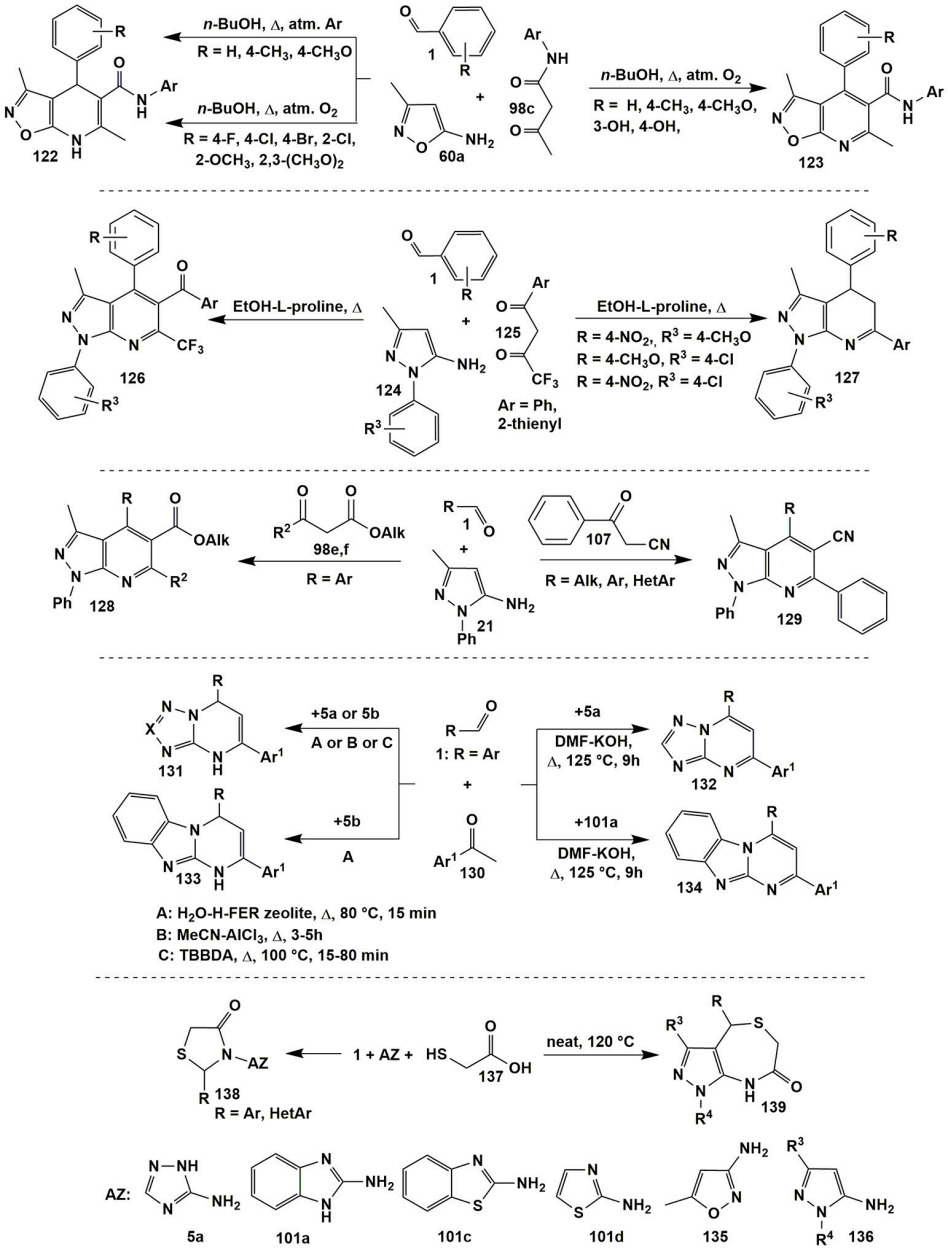
MCR*s* involving α-aminoazoles, aldehydes and non-cyclic carbonyl compounds.

Steric and electronic effects of a substituent R in aldehyde **1** significantly influenced the ability to oxidation of dihydropyrimidine cycle in the condensations involving 5-amino-3-methylisoxazole (**60a**) and aromatic aldehydes **1** with *N*-arylacetoacetamides **98c**. Thus, under identical conditions (*n*-BuOH, Δ, oxygen of air) dihydropyridines **122** were isolated only in case of *para*-halogeno- and *ortho*-substituted aldehydes **1**. The authors (Tkachenko et al., 2014b) associate this with the electronic influence of the halogenaryl moiety or the steric effect of *ortho*-substituents, which complicates the oxidation of heterocycles **122** to **123**. In case of other aldehydes such conditions led to the formation of heteroaromatized systems **123**. Only carrying out the reaction in the argon atmosphere afforded isolation of dihydropyridines **122** (except hydroxy-substituted ones). However, blowing oxygen through their ethanol solutions led to the transformation of compounds **122** to **123** (Figure 10).

The influence of substituents on the direction of a reaction involving asymmetric 1,3-diketones **125**, aromatic aldehydes **1** and 5-amino-1-aryl-3-methylpyrazoles **124** was also significant. The regioselectivity of the formation of aromatic pyrazolopyridines **126** was caused by a greater electrophilicity of COCF_3_ than COAr-carbonyl group. However, for some combinations of substituents in 5-aminopyrazole **124** and aldehyde **1** dihydropyrazolopyridines **127** without trifluoroacetyl moiety were formed (Figure 10; Gunasekaran et al., 2013).

When 5-amino-3-methyl-1-phenylpyrazole (**21**) reacted with aldehydes **1** and other CH-acids (acetoacetic acid derivatives), e.g., **98e,f** (Fan et al., 2016), 3-oxo-3-phenylpropanenitrile **107** (Huang et al., 2011; Rahmati and Khalesi, 2012) heteroaromatic pyrazolopyridines **128** (similar to compounds **126**) and **129** (similar to compounds **118a**, Figure 8) were formed (Figure 10).

When acetophenones **130** were used as CH-acids in condensations with aldehydes **1** and different aminoazoles [3-amino-1,2,4-triazole (**5a**), 5-aminotetrazole (**5b**), 2-aminobenzimidazole (**101a**), 5-aminopyrazole and 3-aminoindazole] two types of products were formed–azolopyrimidines of types **132** (Palaniraja et al., 2016a) and **134** (Palaniraja et al., 2016a) or their dihydroanalogues **131** (Ghorbani-Vaghei et al., 2013; Hassaneen and Farghaly, 2015; Kour et al., 2017) and **133** (Hassaneen and Farghaly, 2015; Figure 10).

Conditions for the obtaining thiazolidin-4-ones **138** from aldehydes **1**, different aminoazoles and thioglycolic acid (**137**) were dependent on the origin of aminoazole. Thus, for 3-amino-1,2,4-triazole **5a** (Ebrahimi, 2016) the cyclization was performed under solvent-free conditions with addition of ammonium persulfate as a catalyst (Δ, 90°C, 1 h); for 2-aminobenzimidazole (**101a**) (Kumar et al., 2013) and 2-aminobenzothiazole (**101c**) (Kumar et al., 2013)–in toluene with addition of HClO_4_-SiO_2_ catalyst (Δ, 100°C, 3–6 h); for 2-aminothiazole (**101d**) (Wu et al., 2014)–in toluene (Δ, 140°C) and for 3-amino-5-methylisoxazole (**135**) (Murugesan et al., 2014)–in toluene (Δ, 140°C, ~24 h). Application of 1- and 3-substituted 5-aminopyrazoles **136** (Abonia, 2014) with the reagents **1** and **137** under solvent-free conditions and self-catalysis with thioglycolic acid (**137**) afforded cyclic pyrazolo[3,4-*e*][1,4]thiazepin-7(4*H*)-ones **139** to be isolated (Figure 10).

A special attention should be also paid to the multicomponent reactions of aminoazoles and aromatic aldehydes with pyruvic acid and its derivatives, especially because of the ambiguity in the realization of directions of such processes. Chebanov's group (Chebanov et al., 2005, 2007a, 2012a; Sakhno et al., 2008, 2010, 2011, 2015) contributed a lot to studying both stepwise and MCR reactions involving pyruvic acid and aminoazoles and showed that their chemo- and regioselectivity, positional orientation of the substituents in the final products significantly depend on the reaction parameters and structure of the starting reagents.

**Figure 11:**
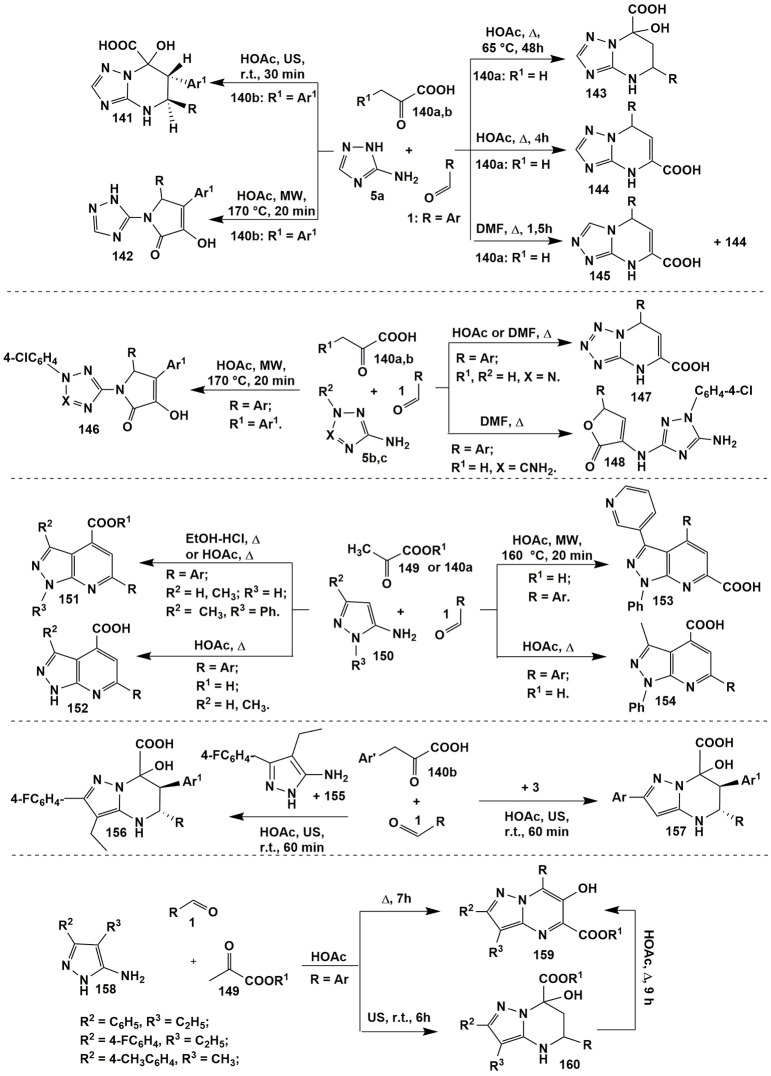
MCR*s* involving aminoazoles, aldehydes and pyruvic acid derivatives.

Thus, in the heterocyclizations involving 3-amino-1,2,4-triazole (**5a**) and aromatic aldehydes **1** with pyruvic acid (**140a**) (R^1^ = H) dihydrotriazolopyrimidines **144** (Chebanov et al., 2005) with the same positional orientation as for acetoacetic acid reaction (heterocycle **100**, Figure 8) were formed (HOAc, Δ, 4 h). When compounds **1**, **5a**, and **140a** were refluxed in DMF dihydrotriazolopyrimidine **144** was obtained in a mixture with regioisomer **145** (which was impossible to isolate in a pure state). Later on it was found that prolonged heating of compounds **1**, **5a**, and **140a** (HOAc, Δ, 65°C, 48 h) also afforded tetrahydroderivatives **143** (Murlykina et al., 2015) that could be converted into dihydropyrimidines **144** after refluxing in HOAc for 4 h (Figure 11).

Temperature regime was also crucial in the condensations involving arylpyruvic acid **140b** (R^1^ = Ar): (Sakhno et al., 2008, 2010; Murlykina et al., 2015), tetrahydropyrimidines **141** were yielded under the room temperature conditions (HOAc, US, r.t., 30 min) while pyrrolones **142** were obtained at elevated temperatures (HOAc, MW, 170°C, 20 min). Authors also carried out the transformation of compounds **141** into **142** (HOAc, MW, 170°C, 40 min) and proved the formation of compounds **141** and **142** under kinetic or thermodynamic control, respectively (Figure 11).

Heterocyclizations involving 5-aminopyrazolecarboxamides (Chebanov et al., 2007a) under the same conditions afforded products of types **141**, **142** and **144** that again indicated on the similar behavior of substituted in the position 4 pyrazoles and 3-amino-1,2,4-triazole that had been already mentioned for the condensations with acetoacetic acid derivatives (see Figure 8).

In the contrary, the behavior of 5-aminotetrazole (**5b**) (X = N, R^2^ = H) and 1-(4-chlorophenyl)-3,5-diamino-1,2,4-triazole (**5c**) (X = CNH_2_, R^2^ = 4-ClC_6_H_4_) was somewhat different. Application of the same conditions (HOAc or DMF, Δ) or (EtOAc-I_2_, Δ) (Zeng et al., 2011) for the condensation of aldehydes **1**, pyruvic acid (**140a**) and 5-aminotetrazole (**5b**) gave the analogous to compounds **144** dihydrotetrazolopyrimidines **147** (Chebanov et al., 2005) while in case of 1-(4-chlorophenyl)-3,5-diamino-1,2,4-triazole (**5c)**–furanones **148** (Sakhno et al., 2011). Pyrrolones **146** (Sakhno et al., 2008) were the high-temperature products of the condensations involving both aminoazoles **5b** and **5c** with arylpyruvic acids [as in case of 3-amino-1,2,4-triazole (**5a**)]. All the attempts to isolate dihydropyrimidine acids of type **147** on the basis of 3,5-diaminotriazole **5c** were unsuccessful that was explained (Sakhno et al., 2011) by the loss of aromaticity in azole cycle during the formation of the fused fragment (Figure 11).

A large number of pyrrolones was also synthesized by Ryabukhin et al. (2012) in the reactions of ethyl 2,4-dioxo-4-arylbutanoates with aldehydes and 2-aminobenzothiazole, 2-aminothiazole, 2-amino-1,3,4-thiadiazole, 5-amino-1,2,4-thiadiazole, 3-amino-5-methylisoxazole, 3-amino-4-methyl-1,2,5-oxadiazole.

Condensation of pyruvic acid (**140a**) or its esters **149** and aldehydes **1** with 5-amino-3-aryl(alkyl)pyrazoles **150** afforded heteroaromatic pyrazolopyridine acids **152** (Cowen et al., 2016) and **154** (Chebanov et al., 2007a) (HOAc, Δ), their esters **151** [HOAc, Δ (Chebanov et al., 2007a) or EtOH-HCl, Δ (Dias et al., 2007)], correspondingly. It's interesting to note, that in case of 5-amino-1-phenyl-3-(pyridine-3-yl)-1*H*-pyrazole El-Borai et al. (2012) obtained pyrazolopyridines **153** (HOAc, MW, 160°C, 20 min) with different positional orientation of substituents than in the products **151**, **152**, **154** (Figure 11).

Introduction of arylpyruvic acid **140b** into the same high-temperature reaction yielded no pyrrolone of type **142** but led to the tarring the reaction mixture. Only reducing temperature together with applying ultrasonic activation allowed to synthesize tetrahydropyrazolopyrimidines **156** and **157** (Murlykina et al., 2013; similar to compounds **141**) in cases of 4-ethyl-5-amino-3-(4-fluorophenyl)pyrazole **155** and 5-amino-3-aryl-pyrazole **3** (HOAc, US, r.t., 60 min; Figure 11).

The analogous tetrahydropyrazolopyrimidines **160** (Sakhno et al., 2018) were synthesized in the reaction of 3-aryl-4-alkyl-substituted 5-aminopyrazoles **158** and aromatic aldehydes **1** with alkyl pyruvates **149** in acetic acid at room temperature. At the same time refluxing compounds **1**, **149** and **158** in acetic acid for 7 h led to the formation of a pyrimidine ring followed by an oxidative heteroaromatization process which gave 6-hydroxy-substituted alkyl pyrazolopyrimidine-5-carboxylates **159** (Sakhno et al., 2018). That was explained by disproportionation process; it was confirmed by carrying out this reaction in the inert atmosphere (where neither dihydropyrazolopyrimidine nor the compound without a hydroxyl group was observed). As it was expected, heterocycles **160** were transformed into heteroaromatic derivatives **159** upon boiling in acetic acid for 9 h (Figure 11).

**Figure 12:**
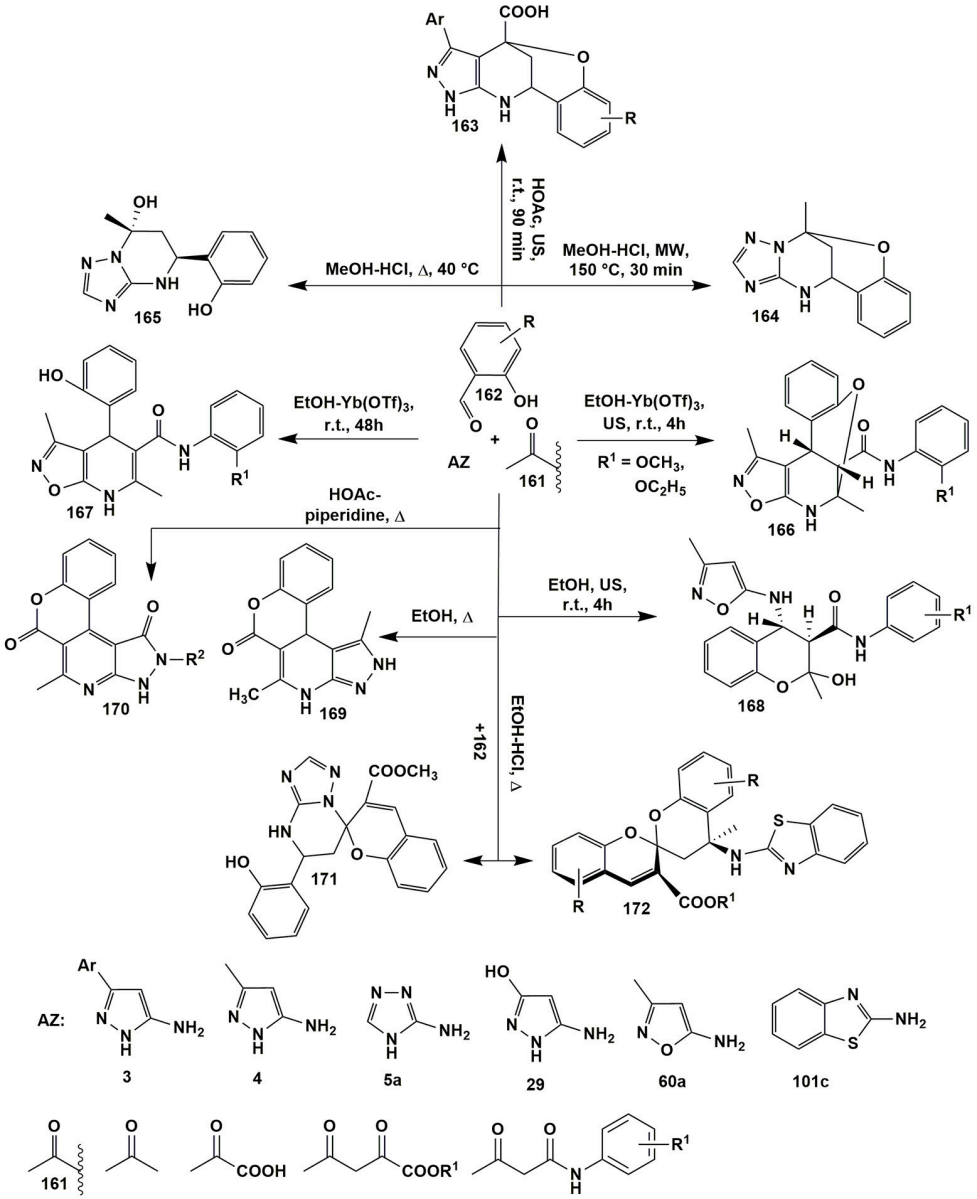
Some post-cyclizations serving as an additional source of molecular diversity in MCRs involving salicylic aldehydes, aminoazoles and active methylene compounds.

Post-cyclizations can serve as an additional source of molecular diversity in the MCR*s*. They occur, for example, when salicylaldehyde is used in the MCR*s*. Thus, Gorobets et al. (2010) by varying temperature in the MCR of salicylaldehyde **162** with acetone (**161**) and 3-amino-1,2,4-triazole (**5a**) yielded both bridged benzoxadiazocines **164** (MeOH-HCl, MW, 150°C, 30 min) and tetrahydroderivatives **165** (CH_3_OH-HCl, Δ, 40°C). Later on several groups (Kondratiuk et al., 2016; Gümüş et al., 2017a; Komykhov et al., 2017; Aydemir et al., 2018) studied the aspects of these transformations in details. It's interesting, that condensation involving 5-amino-3-arylpyrazoles **3**, salicylaldehydes **162** and pyruvic acid (**140a**), unlike the MCR with 3-amino-1,2,4-triazole (**5a**), afforded bridged benzoxazocines **163** at room temperature (HOAc, US, 90 min) whereas microwave heating at 150°C led to heteroaromatized pyrazolopyridines of type **152** (Figure 12).

Varying the conditions of the reaction and structures of the starting reagents afforded to synthesize three different classes of compounds **166**–**168** from the same reagents (Tkachenko et al., 2014a). Ultrasonication of 5-amino-3-methylisoxazole (**60**), *N*-aryl-3-oxobutanamide (**161**) and salicylaldehyde (**162**) afforded *N*-aryl-4-(3-methylisoxazole-5-ylamino)chromane-3-carboxamides **168**. Stirring *N*-aryl-3-oxobutanamides **161** with R^1^ = 2-CH_3_O- or 2-C_2_H_5_O- and compounds **162** and **60** in the presence of Yt(OTf)_3_ redirected the condensation toward the formation of dihydroisoxazolopyridines **167**, whereas ultrasonication led to benzoxazocines **166**. This was almost an exceptional case when the replacement of the usual stirring by ultrasonic activation under other identical conditions led to the formation of different compounds (Figure 12; Tkachenko et al., 2014a).

It's worth to note, that in case of other substituted *N*-aryl-3-oxobutanamides **161** (R^1^ = 2-OH, 2-CH_3_, 2-Cl, 3-Cl) only heterocycles **167** were isolated both with the help of mechanical stirring and under ultrasonication. The authors (Tkachenko et al., 2014a) suppose that the direction leading to benzoxazocines **166** is favored by the formation of 3-coordinated complex of Yt(OTf)_3_ with NH- and CH_3_O(C_2_H_5_O)-groups of carboxamide fragment and OH-group of intermediate tetrahydroisoxazolopyridine. In turn, ultrasound supplies to the reaction system a sufficient amount of energy that is needed for nucleophilic substitution and bridged moiety formation (Figure 12).

*o-*Hydroxyl group of aldehyde **162** can also participate in the formation of lactones of types **169** (Svetlik et al., 2010) and **170** (Frolova et al., 2011), that were synthesized in the condensation of salicylaldehyde (**162**), 5-aminopyrazoles **4** or **29** and acetoacetic acid esters **161**. Heterocyclization of compound **161** with a double excess of salicylaldehyde (**162**) and 3-amino-1,2,4-triazole (**5a**) or 2-aminobenzothiazole (**101c**) gave spiroheterocycles **171** (Světlík and Kettmann, 2011) and **172** (Svetlík et al., 2014); 2-aminobenzimidazole reacted with an exocyclic amino-group as a mononucleophile (Figure 12).

**Figure 13:**
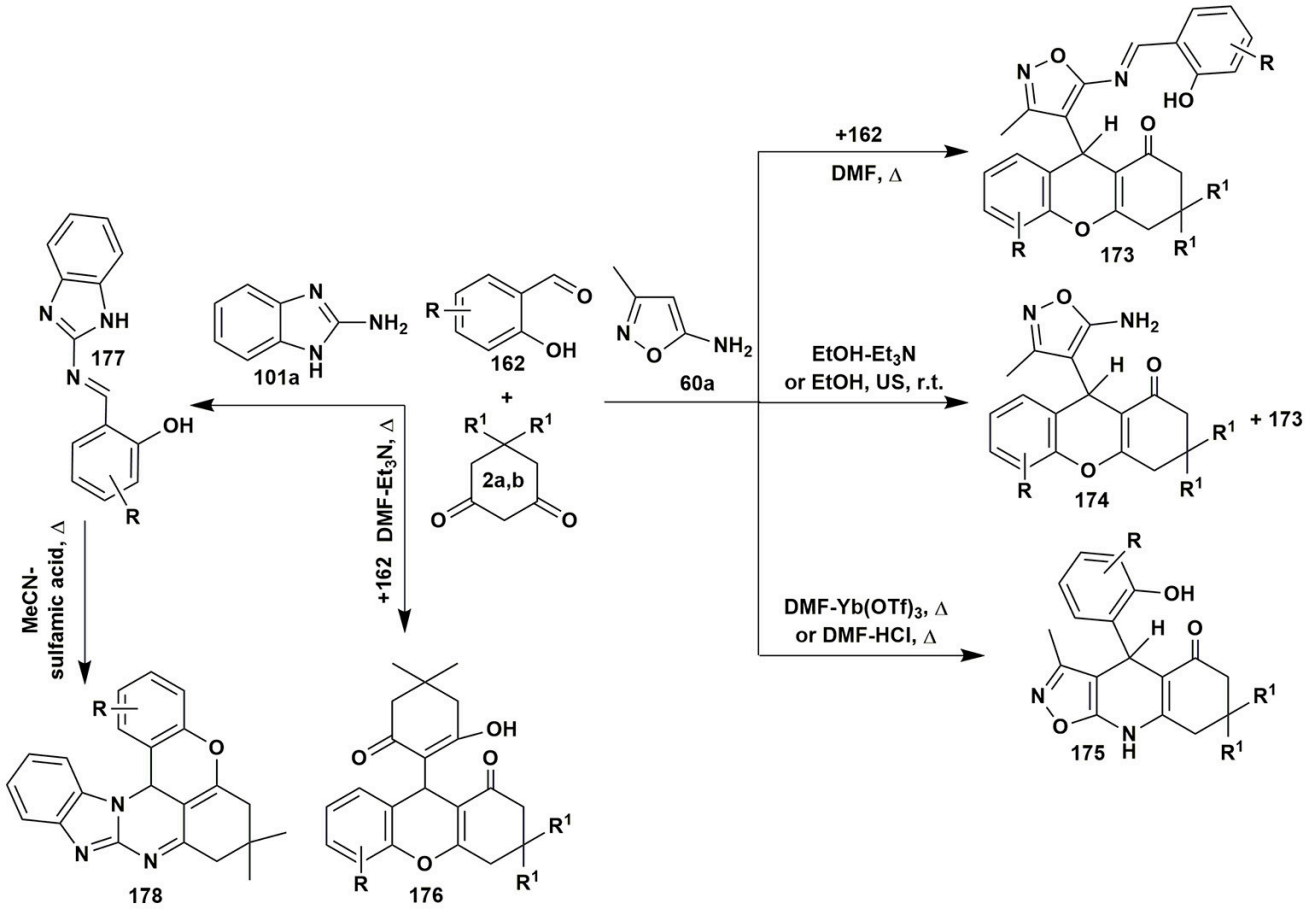
Condition-based divergence strategy in MCR*s* of aminoazoles with salicylic aldehydes and cyclic active methylene compounds.

In the multicomponent reaction of 5-amino-3-methylisoxazole (**60a**) and salicylaldehydes **162** with 1,3-cyclohexanediones **2** Muravyova et al. (2013) synthesized several heterocycles **173**–**175** depending on the conditions. In case of 2-aminobenzimidazole (**101a**) condensation with the reagents **2** and **162** under the different conditions (heating in toluene with addition of K_2_CO_3_ or heating in chloroform with addition of sulfamic acid) resulted exclusively in xanthene-1-ones **176**. Only the stepwise transformation involving the preliminary synthetized imine **177** with dimedone (**2b**) afforded tetrahydrobenzo[4,5]imidazo[2,1-*b*]chromeno[4,3,2-*de*]quinazolines **178** (Saeedi et al., 2011; Figure 13).

### Other multicomponent reactions of aminoazoles

**Figure 14:**
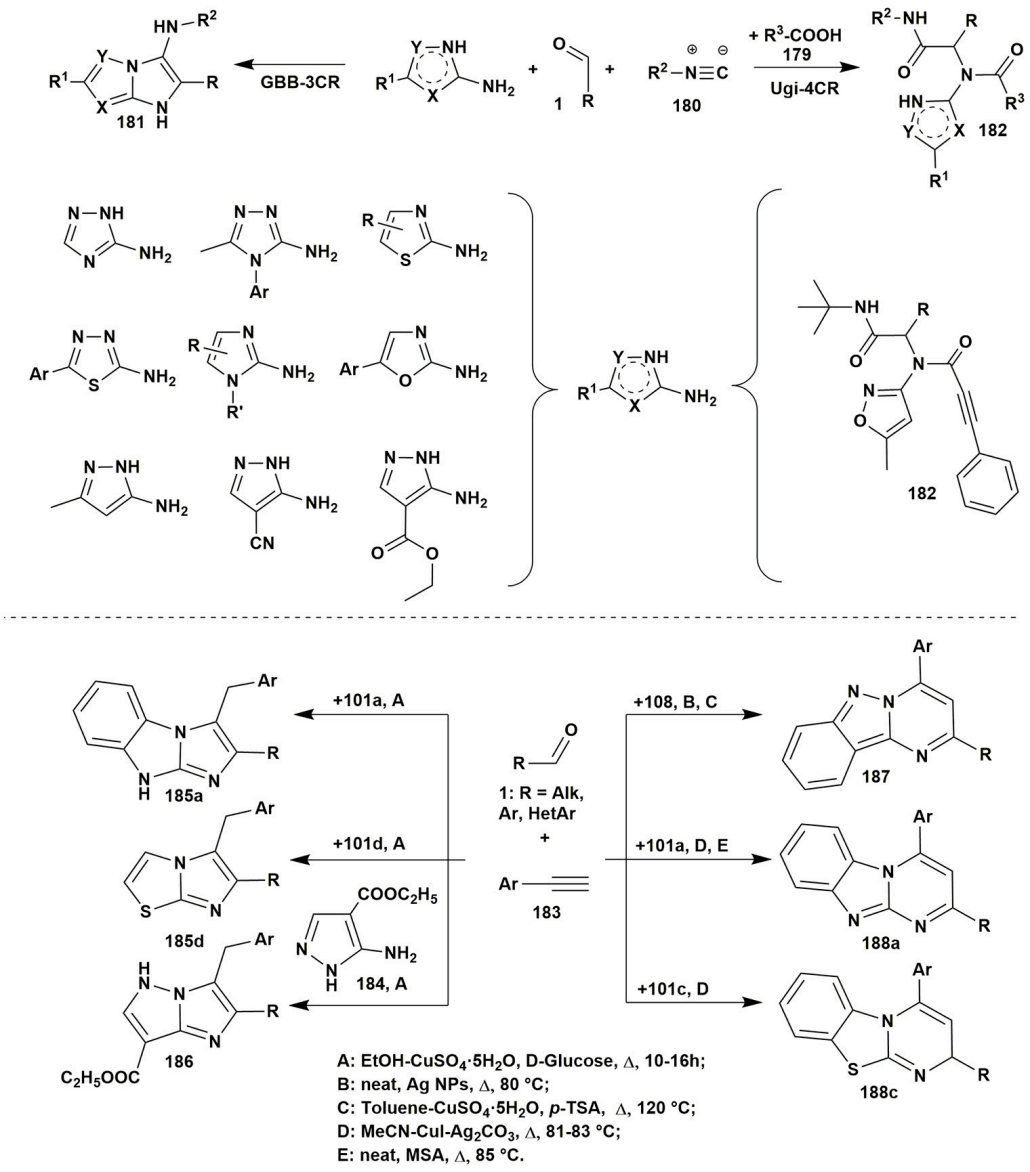
Examples of the application of aminoazoles in GBB-3CR, Ugi-4CR and A^3^ coupling reactions.

Isocyanide-based reactions may be separated into an individual large group and certainly should be described in special reviews, a lot of brilliant examples of which have already been published (Dömling and Ugi, 2000; Banfi et al., 2010; Ruijter et al., 2011; Dömling et al., 2012; Cioc et al., 2014; Koopmanschap et al., 2014; Devi et al., 2015; Zarganes-Tzitzikas et al., 2015; Shaaban and Abdel-Wahab, 2016). As it's recognized the classical components of the Ugi four-component reaction (Ugi-4CR) are aliphatic or aromatic amines and aldehydes, carboxylic acids and substituted isocyanides, that are generally well responsive to the formation of Ugi products at room or slightly elevated temperatures (Dömling and Ugi, 2000; Dömling, 2006). Groebke-Blackburn-Bienaymé three-component reaction (GBB-3CR) usually undergoes with participation of 2-aminoazines or 2-aminoazoles, aromatic or aliphatic aldehydes and substituted isocyanides. Brønsted or Lewis acids are often used in GBB-3CR (sometimes in Ugi-4CR) for activation of intermediate imine. Almost all the types of solvents (including water and ionic liquids) and catalysts, different temperature regimes (conventional or microwave heating) were studied in GBB-3CR (Figure 14; Devi et al., 2015).

There are a lot of examples of using aminoazoles as an amine component in GBB-3CR resulting in the formation of heterocycles like **181**. The most studied ones are the processes involving 3-amino-1,2,4-triazoles (Bienaymé and Bouzid, 1998; Tyagi et al., 2012; Urich et al., 2013; Aouali et al., 2015), 2-amino(benzo)thiazoles (Bienaymé and Bouzid, 1998; Guchhait and Madaan, 2009, 2010; Guchhait et al., 2009; Al-Tel et al., 2010; Akritopoulou-Zanze et al., 2011; Baviskar et al., 2011; Burchak et al., 2011; Hieke et al., 2012; Tyagi et al., 2012; Vidyacharan et al., 2014; Martinez-Ariza et al., 2015; Ansari et al., 2016; Shaabani and Hooshmand, 2016; Shao et al., 2017), 2-amino-1,3,4-thiadiazoles (Krasavin et al., 2008; Guchhait and Madaan, 2009; Guchhait et al., 2009; Wadhwa et al., 2015), 2-amino(benz)imidazoles (Lee et al., 2013; Pereshivko et al., 2013). GBB-3CR involving 2-aminooxazoles (Bienaymé and Bouzid, 1998) led to the formation of imidazoazoles while involving 1,2,5-oxadiazole-3,4-diamine (Kysil et al., 2010) gave oxadiazolopyrazines. Groebke condensations of 5-aminopyrazoles (5-amino-3-methylpyrazole, 5-aminopyrazole-4-carbonitrile, ethyl 5-aminopyrazole-4-carboxylate) are described in the following publications (Bienaymé and Bouzid, 1998; Guchhait and Madaan, 2009; Guchhait et al., 2009; Baviskar et al., 2011; Rahmati and Kouzehrash, 2011; Rahmati et al., 2013; Demjén et al., 2014; Murlykina et al., 2017).

Although there are a lot of variations and modifications known for the Ugi-4CR there is only one example of using aminoazole as an amine component in this reaction, which includes the treatment of 3-amino-5-methylisoxazole, aromatic aldehydes, phenylpropiolic acid and *tert*-butylisocyanide with formation of Ugi-product **182** (Figure 14; Murlykina et al., 2017).

Several publications deal with A^3^ coupling reactions between aromatic aldehydes **1**, aryl acetylenes **183** and aminoazoles resulting in the formation of two types of products–via multicomponent assembly reaction through 6-endo-dig (heterocycles **187**, **188**) or through 5-exo-dig cyclization (heterocycles **185**, **186**). Thus, imidazoazoles **185a,d** and **186** were synthesized via method A [EtOH-CuSO_4_·5H_2_O, D-glucose, Δ, 10–16 h (Guchhait et al., 2012)] on the basis of 2-aminobenzimidazole (**101a**), 2-aminothiazole (**101d**) and ethyl 5-aminopyrazole-4-carboxylate (**184**) via A^3^-coupling reaction followed with 5-exo-dig cycloisomerization and prototropic shift (Figure 14).

**Figure 15:**
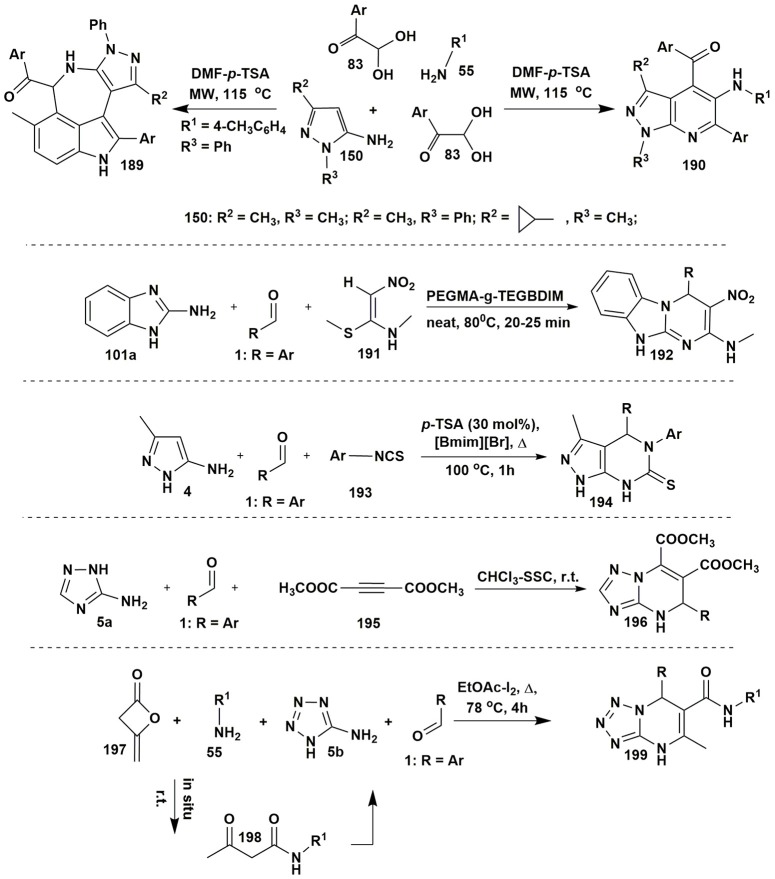
Examples of other MCRs involving aminoazoles.

At the same time, coupling involving 2-aminobenzimidazole (**101a**) (or 2-aminobenzothiazole **101c**) under other conditions [MeCN-CuI-Ag_2_CO_3_, Δ, 81–83°C (Kumar et al., 2014)] or [neat, MSA (molybdate sulfuric acid), Δ, 85°C (Shinde and Jeong, 2015)] led to formation of the benzimidazolopyrimidines **188a** (or benzothiazolopyrimidines **188c**). Carrying out the reaction involving 1*H*-indazol-3-amine (**108**) under neat conditions [Ag NPs (nanoparticles), Δ, 80°C (Balwe et al., 2017)] or in toluene [CuSO_4_·5H_2_O, *p*-TSA Δ, 120°C (Palaniraja et al., 2016b)] resulted in multicomponent assembly reaction through 6-endo-dig cyclization and formation of pyrimido[1,2-*b*]indazoles **187** (Figure 15).

A four-component strategy for the selective synthesis of fused azepino[5,4,3-*cd*]indoles **189** and pyrazolo[3,4-*b*]pyridines **190** was elaborated by Jiang et al. (2014). The direction of multicomponent reaction involving 1,3-substituted 5-aminopyrazoles **150** and amines **55** with two equivalents of arylglyoxals **83** (DMF-*p*-TSA, MW, 115°C) was dependent on the electronic effects of arylglyoxals and aromatic amines (Figure 15).

Reddy et al. (2017) developed a highly active and stable heterogeneous POEGMA-g-TEGBDIM (polyethylene glycol methacrylate-grafted tetraethyleneglycol-bridged dicationic imidazolium-based IL) catalyst for the synthesis of substituted benzo[4,5]imidazo[1,2-*a*]pyrimidine heterocycles **192** upon solvent-free conditions (80°C) from 2-aminobenzimidazole (**101c**), aromatic aldehydes **1** and (*E*)-*N*-methyl-1-(methylthio)-2-nitroethenamine (**191**) (Figure 15).

A synthetic pathway to access fused pyrazolo[3,4-*d*]pyrimidine-6(7*H*)-thiones **194** by the three-component reaction of 5-amino-3-methylpyrazole (**4**), aldehydes **1** and arylisothiocyanates **193** in an ionic liquid in the presence of catalytic amounts of *p*-TSA was elaborated by Safaei et al. (2012; Figure 15).

Application of heterogeneous solid base, silica sodium carbonate (SSC) as a catalyst allowed isolation of dimethyl 4,5-dihydrotriazolopyrimidine-6,7-dicarboxylates **196** in the MCR of dimethyl acetylenedicarboxylate (**195**) with **1** and **5a**. The authors (Karami et al., 2015b) suggested that the base favors the formation of an intermediate product of condensation between nucleophilic NH in the position 2 of 3-amino-1,2,4-triazole (**5a**) and electrophilic CH-center of dimethyl acetylenedicarboxylate (**195**) followed by the attack of aldehyde **1**, cyclization and dehydration (Figure 15).

In some cases, synthesis of starting materials for MCRs is also a difficult task. For example, the formation of acetoacetamide building-block by synthetic methods is an expensive and difficult procedure. Therefore, to avoid laborious stage of acetoacetamide synthesis, as a continuation of the work of Shaabani et al. (2009) four-component procedure for obtaining *N*,7-disubstituted-5-methyl-4,7-dihydrotetrazolo[1,5-*a*]pyrimidine-6-carboxamides **199** (Zeng et al., 2012) was elaborated. It consisted of the reaction of primary amines **198**, diketene **197**, 5-aminotetrazole (**5b**) and aldehydes **1** (EtOAc-I_2_, Δ, 78°C, 4 h). In this MCR acetoacetamide was formed *in situ* by the addition of amine to diketene molecule (Figure 15).

### Two-component condensations of aminoazoles

**Figure 16:**
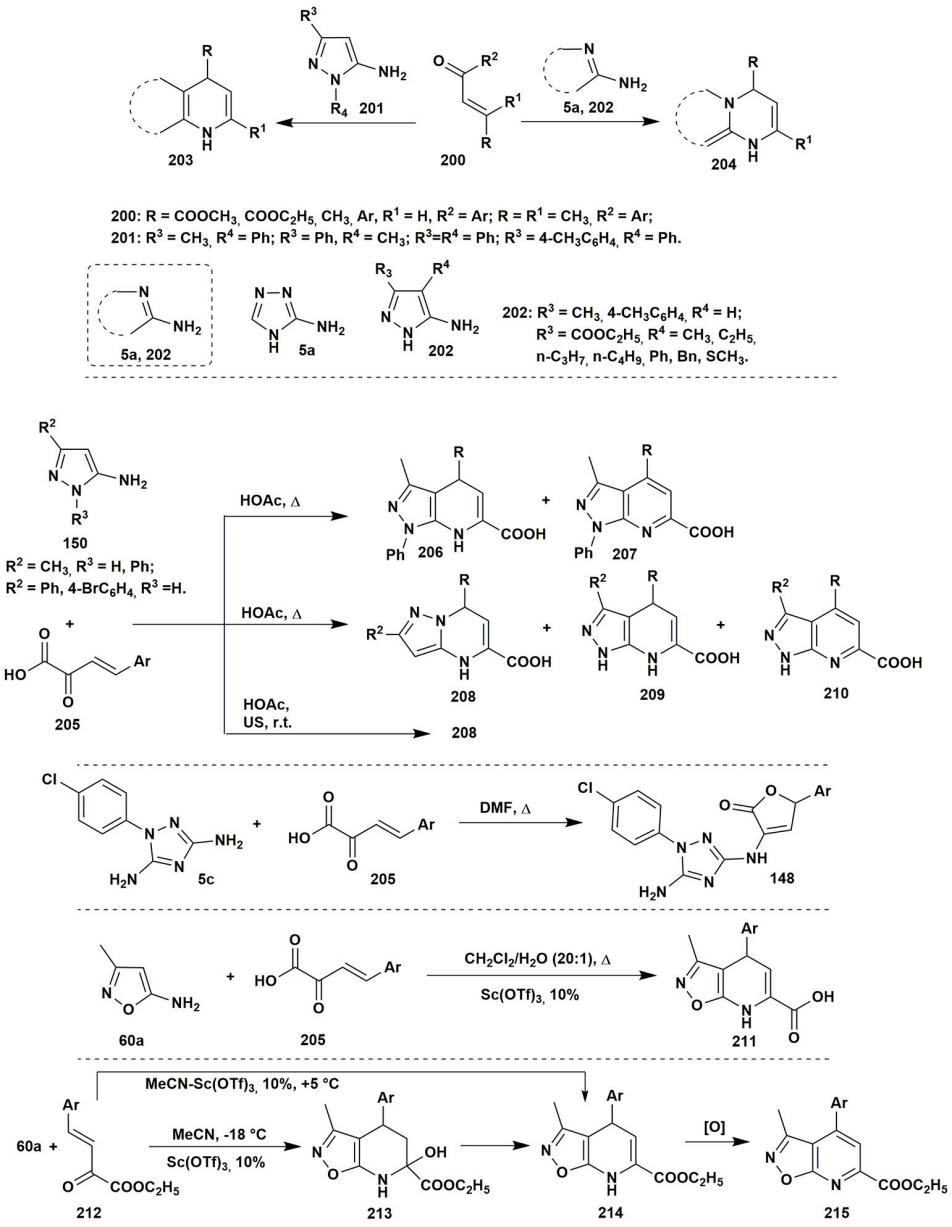
Examples of two-component heterocyclizations of the aminoazoles with α,β-unsaturated carbonyl compounds.

To the best of our knowledge the condensations of aminoazoles with α,β-unsaturated carbonyl compounds **200** could be performed as one of the simplest and effective ways to the diverse azoloazine systems, such as **203**, **204**, since this type of starting materials usually contains alternative nucleophilic and electrophilic reaction centers. The most utilized α,β-unsaturated carbonyl compounds in such reactions are chalcones or cinnamic acid derivatives. The condensations of the enones with aminoazoles could be performed in various solvents within wide range of the temperatures and with application of different types of catalysis (Kolos et al., 2011; Yoshida et al., 2011; Orlov and Sidorenko, 2012; Figure 16).

Two-component heterocyclizations of the aminoazoles could be considered as convergent procedures concerning the corresponding multicomponent synthesis, or as independent transformations. Thus, in the previous section of the review it was shown that multicomponent heterocyclizations of the pyruvic acid derivatives with α-aminoazoles and carbonyl compounds could be applied for the synthesis of diverse heterocyclic systems. However, preliminary condensation of the pyruvic acid with aromatic aldehyde gives arylidenepyruvic acids **205** and their further reaction with 5-aminopyrazoles **150** in comparison to the multicomponent procedure allows to obtain different regioisomers **208**, **209** (Chebanov et al., 2007a, 2012a). At the same time, in the article (Sakhno et al., 2011) it was shown that the two-component condensation of arylidenepyruvic acid **205** and 1-(4-chlorophenyl)-3,5-diamino-1,2,4-triazole (**5c**) in DMF resulted in the formation of the same furanones **148** as in the corresponding MCR, however, in smaller yields (Figure 16).

The opposite pattern was observed in case of the 5-amino-3-methylisoxazole (**60a**) (Morozova et al., 2016). Multicomponent condensation of this aminoazole, pyruvic acid and aromatic aldehyde resulted in the decomposition of the initial amine due to the low stability of the isoxazole moiety in the acidic media. Application of the two-component procedure, via preliminary synthesis of unsaturated acids **205**, under Sc(OTf)_3_ catalysis in CH_2_Cl_2_:H_2_O (20:1) allowed to isolate compound **211** in low yields. An unexpected result was obtained when the unsaturated acid was replaced by the corresponding ethyl ester **212**: the condensation of the starting reagents in MeCN containing Sc(OTf)_3_ at −18°C resulted in the formation of tetrahydroisoxazolopyridine system **213**. Typically, such compounds cannot be isolated due to the fast water elimination with the formation of dihydropyridine rings. Indeed, the condensation at higher temperatures led to the formation of dihydropyridine **214**, which was further spontaneously oxidized (Figure 16).

**Figure 17:**
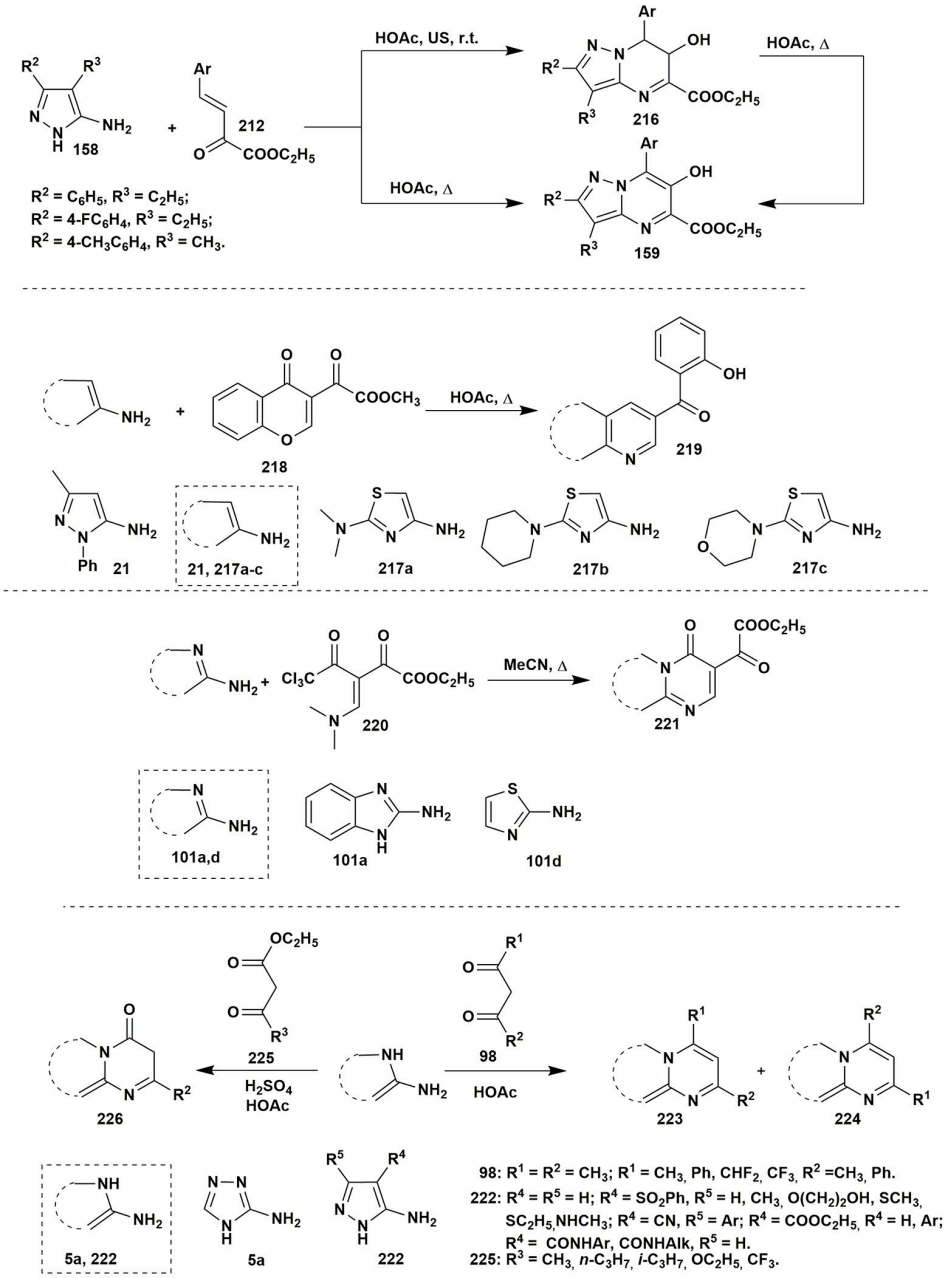
Examples of the application of 1,3-dielectrophiles in the azoloazine synthesis.

The condensation of ethyl arylidenepyruvate **212** with 5-aminopyrazoles **158** in acetic acid without additional catalyst (Sakhno et al., 2018) had a different character and allowed to isolate both pyrazolopyrimidines **159** (under heating) and dihydropyrazolopyrimidines **216** (at room temperature) having OH-group in position 6 (Figure 17). The yields of the product **159** for this two-component condensation were better in comparison to the multicomponent procedure (Figure 11).

3-Methoxalylchromone (**218**) containing hidden α,β-unsaturated fragment exhibited properties being similar to the derivatives of arylidenepyruvic acid: its condensation with different α-aminoazoles **21, 217a–c** resulted in the formation of azoloazines **219** under refluxing in acetic acid or heating in DMF-TMSCl at 80–100°C (Mkrtchyan et al., 2010). Another group studied the condensation of ethyl 5,5,5-trichloro-3-[(dimethylamino)methylene]-2,4-dioxopentanoate (**220**) with 2-aminothiazole or 2-aminobenzimidazole **101a,d**. The starting β-enaminodiketone has two nonequivalent carbonyl groups; however, the condensation with aminoazole is selective through the influence of the trichloromethyl group adjacent to the reaction's site. Nucleophilic attack of the carbonyl carbon atom by the lone electron pair of the endocyclic nitrogen resulted in the elimination of the trichloromethyl group and in the formation of corresponding thiazolo[3,2-*a*]pyrimidinone and pyrimido[1,2-*a*]benzimidazole **221** (Campos et al., 2017; Figure 17).

The application of 1,3-dielectrophiles in the azoloazine synthesis is not limited to the enones. β-Dicarbonyl compounds, for example, derivatives of acetylacetone **98** and acetoacetate **225** (Marjani et al., 2015) are used for the formation of the pyrimidine ring with substituents in positions 4 and 6. The asymmetric β-dicarbonyl compounds can produce positional isomers, but often the reactions give only one compound. The aminoazoles with pyrrole N-atom in the α-position to the NH_2_-group are most often used as 1,3-binucleophiles (Gujjar et al., 2011; Ivachtchenko et al., 2011; Gege et al., 2012; Patnaik et al., 2012; Figure 17).

**Figure 18:**
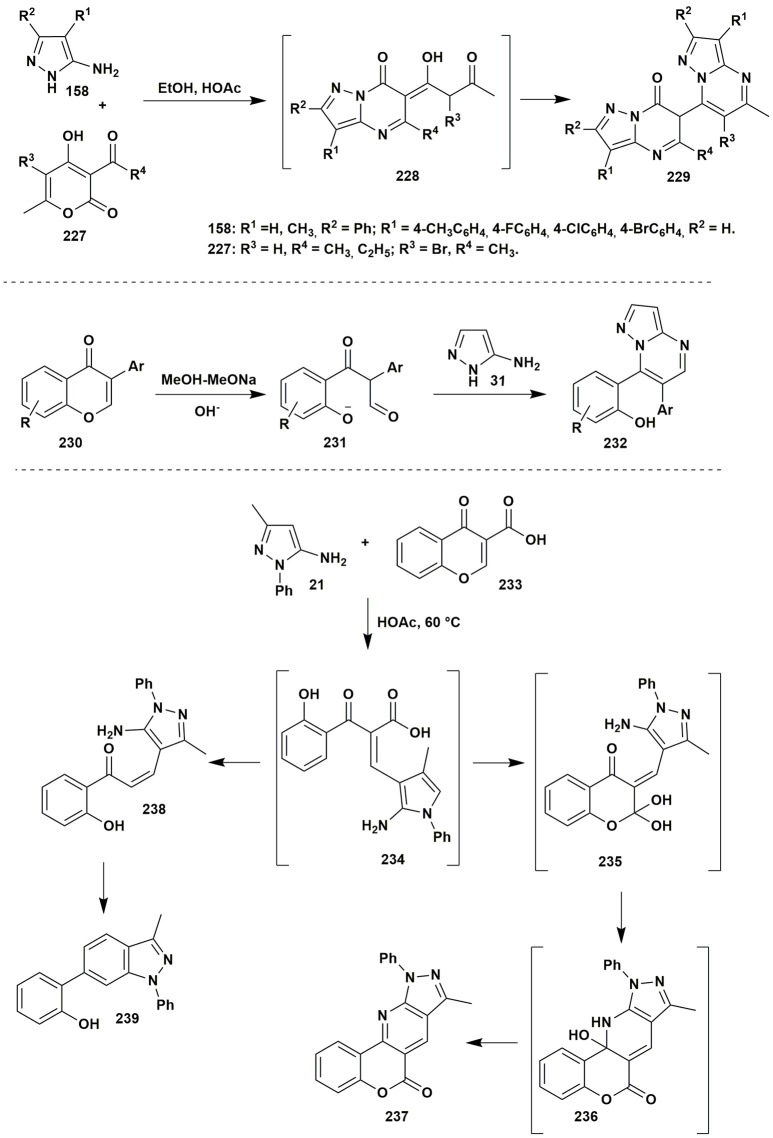
Two-component condensations of aminoazoles with carbonyl compounds containing pyrone moiety.

Among reactions involving β-diketones there is rather interesting condensation of 5-aminopyrazole **158** and dehydroacetic acid **227** (Aggarwal et al., 2014). It was found that the reaction did not stop on the formation of **228**: the presence of the reactive toward 5-aminopyrazole acetylacetone fragment induced further condensation with the second molecule of the amine **158** that gave bis(pyrazolo[1,5-*a*]pyrimidinyl)-7-ones **229** (Figure 18).

Despite the fact that compounds containing 4*H*-chromen-4-one moiety don't have real 1,3-dicarbonyl fragment in the presence of alkali in the reaction mixture the ring-opening process with the generation of the corresponding 1,3-dicarbonyl compound takes place (Zhang et al., 2011). In such way 7-diphenylpyrazolo[1,5-*a*]pyrimidine derivatives **232** were synthesized by the condensation of isoflavone **230** and 3-aminopyrazole (**31**) in MeOH-MeONa in moderate to good yields (Figure 18).

Resembling condensation of chromone-3-carboxylic acid (**233**) and aminopyrazole **21** in acetic acid gave chromeno[4,3-*b*]pyrazolo[4,3-*e*]pyridin-6(10*H*)-one **237** as the major product of the condensation. However, side decarboxylation of the intermediate **234** following with further condensation resulted in the formation of traces of pyrazolo[3,4-*b*]pyridine **239** (Miliutina et al., 2017). The intermediate **238** was isolated in low yields when the reaction was stopped after 1 h at 60°C (Figure 18).

**Figure 19:**
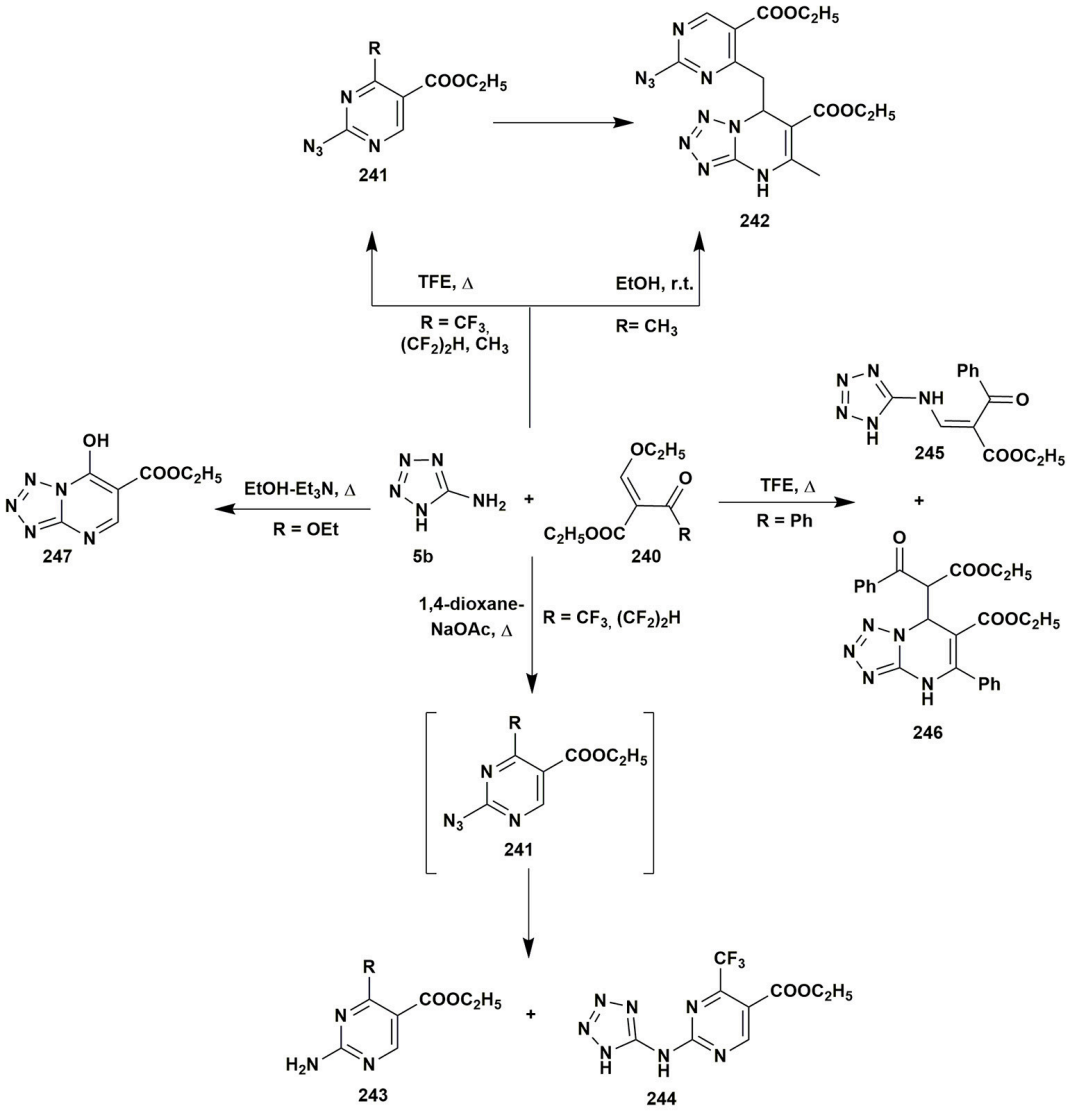
Heterocyclizations of 5-aminotetrazole and 2-ethoxymethylidene-3-oxo esters.

Quite interesting heterocyclizations of 5-aminotetrazole were reported by Goryaeva et al. (2015): heterocyclization of 5-aminotetrazole (**5b**) and 2-ethoxymethylidene-3-oxo esters **240**, depending on the ester type and/or the condition, could give 2-azidopyrimidines **241** or tetrazolo[1,5-*a*]pyrimidines **242**. The starting materials under refluxing in EtOH or 1,4-dioxane didn't react completely even after long duration of the treatment and resulted in the formation of inseparable mixtures. Carrying out the reaction in the 2,2,2-trifluoroethanol (TFE) gave 2-azidopyrimidines **241** due to the opening of the tetrazole ring. At the same time, 4-methyl-2-azidopyrimidine was not stable and converted into the **242** even while standing as solid on air. The synthesis of the substance **242** could be carried out in EtOH at r.t. from 5-aminotetrazole (**5b**) and ester **240**, the presence of the **241** was indicated by TLC in the reaction mixture, which allows to assume that the reaction could pass through the formation of azide (Figure 19).

Despite the fact that the condensation of 5-aminotetrazole (**5b**) with the fluorinated reagents in 1,4-dioxane resulted in the mixtures of compounds, the presence of the catalytic amounts of sodium acetate led to the formation of azide **241** with further elimination of the nitrogen and nitrenes that gave ethyl 2-amino-4-(polyfluoroalkyl)pyrimidine-5-carboxylates **243**. In case of the CF_3_-substituted ester the formation of the side product **244** was observed as well. On the other hand, 2-benzoyl-3-ethoxyprop-2-enoate in the condensation with 5-aminotetrazole in TFE under reflux yielded the mixture of compounds **245** and **246** due to the decomposition of the initial ester and the formation of the reacting ethyl benzoylacetate. Application of 2-ethoxymethylidene malonate **240** under refluxing in EtOH allowed to isolate ethyl-7-hydroxytetrazolo[1,5-*a*]pyrimidine-6-carboxylate **247**. Thus, depending on the substituent in 2-ethoxymethylidene-3-oxo esters different tetrazolopyrimidines or pyrimidines were obtained (Figure 19).

**Figure 20:**
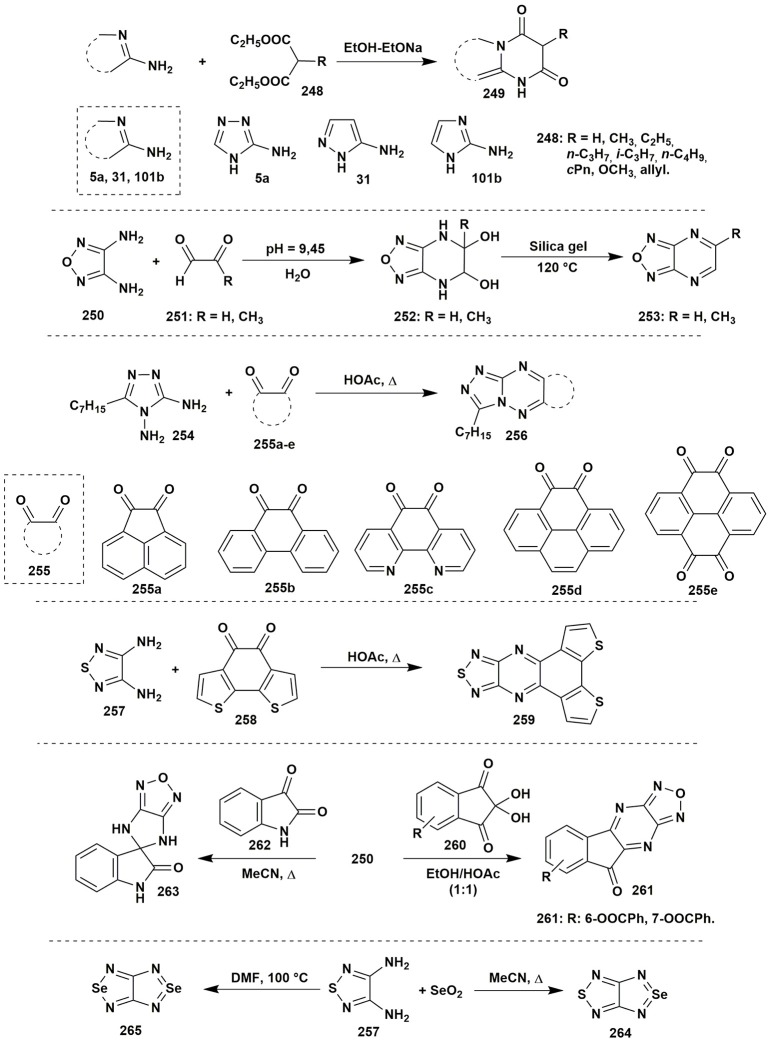
Some two-component transformations of aminoazoles with malonic esters, glyoxals derivatives, hem-diols, isatin, or selenium dioxide.

Acetoacetic esters may be easily replaced by malonic ester or sodium nitromalonaldehyde monohydrate (Ren et al., 2012). The malonic esters **248** were used as efficient starting materials for the synthesis of the azoloazines **249** substituted in the position 5 (Saito et al., 2011; Figure 20).

The condensations of α-dicarbonyl compounds could be carried out with α-diamines as well. An interesting publication was presented by Willer et al. (2012) dealing with short synthetic route to [1,2,5]oxadiazolo[3,4-*b*]pyrazine moiety. The reaction of diamine **250** and glyoxal or pyruvic aldehyde **251** under mild conditions (45°C, 45 min) yielded 5,6-dihydroxy-4,5,6,7-tetrahydro[1,2,5]oxadiazolo[3,4-*b*]pyrazine **252** (R = H; conversion 96%) or its unstable analog **252** (R = CH_3_). Isolation of the pure compound **252** was made by lyophilization or by carrying out the reaction at 20°C. Further pyrolysis on the silica gel gave target [1,2,5]oxadiazolo[3,4-*b*]pyrazines **253** in low yields (10–33%; Figure 20).

Fusco et al. (2016) applied the condensation between α-diamines **254** and α-diketones **255a–e** to obtain triazolo[4,3-*b*][1,2,4]triazines **256** in 25–95% yield (the lowest yield for a system with two diketone moieties). An analogous result was obtained for 1,2,5-thiadiazole-3,4-diamine **257** (Planells et al., 2014; Figure 20).

The article published by Lauro et al. (2017) presented the possibility to apply hem-diols instead of α-diketones. The condensation of amine **250** with the selected 2,2-dihydroxy-1*H*-indene-1,3(2*H*)-diones **260** gave compounds **261** in moderate yields (Figure 20).

Diaminoazoles can be applied not only for synthesis of six-membered heterocycles: the condensation of isatin (**262**) and 1,2,5-oxadiazole-3,4-diamine (**250**) in the boiling acetonitrile gave 4,6-dihydrospiro(imidazo[4,5-*c*][1,2,5]oxadiazol-5,3′-indol)-2′(1′*H*)-one **263** (Gurevich et al., 2011; Figure 20).

Very unusual application of selenium dioxide as a carbonyl compound in the condensation with 1,2,5-thiadiazole-3,4-diamine (**257**) giving [1,2,5]selenadiazolo[3,4-*c*][1,2,5]thiadiazole (**264**) was described (Konstantinova et al., 2015). As the side reaction an exchange of the sulfur of the thiadiazole was observed: that also allowed to isolate [1,2,5]selenadiazolo[3,4-*c*][1,2,5]selenadiazole (**265**). The formation of compound **265** as the major product of the heterocyclization could happen in DMF at 100°C (Figure 20).

**Figure 21:**
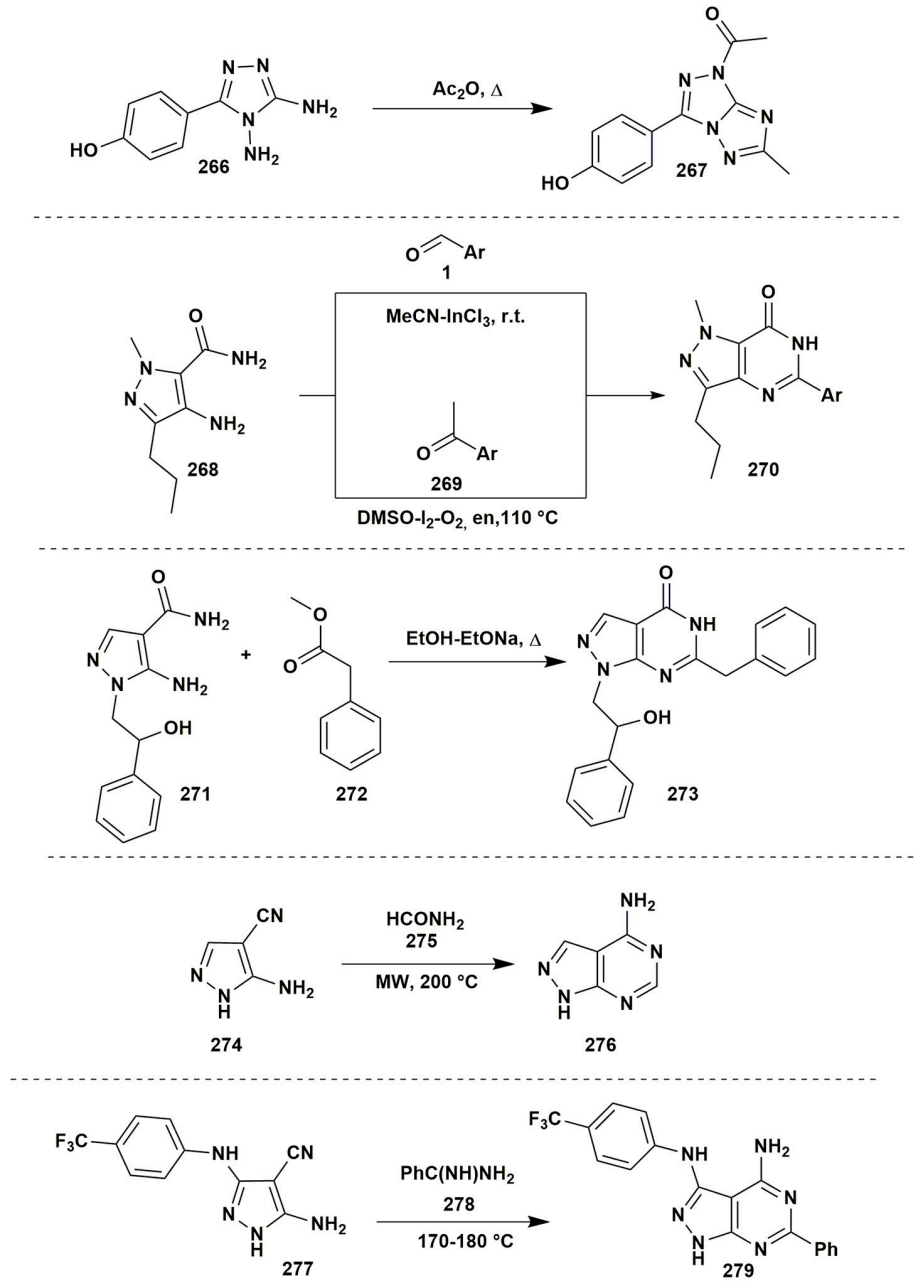
The condensations of aminoazoles having additional nuclephilic reaction center.

The acylation of the vicinal diamines by the acetic anhydride under reflux could give the new azole ring. Thus, Centore et al. (2017) applied the reaction of 3,4-diamino-1,2,4-triazole (**266**) with acetic anhydride to obtain [1,2,4]triazolo[3,2-*c*][1,2,4]triazole (**267**) without isolation of non-cyclic products of acylation (Figure 21).

The condensations of aminoazoles with carbonyl compounds are not limited to the vicinal amines. The azoles with an amide group next to the amine one also can be used as the reagents for the synthesis of pyrimidinones, but the condensation should be promoted by catalysts. Mulakayala et al. (2012) showed that condensation between 4-amino-1*H*-pyrazole-5-carboxamide (**268**) and aromatic aldehydes **1** occured without Lewis acid neither at room temperature nor under refluxing, however, the presence of the catalytic amounts of InCl_3_ promoted the cyclocondensation. Among the studied solvents, the best result was observed in case of MeCN (10% mol of the catalyst) at room temperature. Variation of the solvents (MeOH, *i-*PrOH, EtOAc, CH_2_Cl_2_, CHCl_3_) or Lewis acids (AlCl_3_, TiCl_4_, BF_3_-OEt_2_, FeCl_3_, CuCl_2_) led to the yields decreasing. Later on another method for synthesis of the similar 1,6-dihydro-7*H*-pyrazolo[4,3-*d*]pyrimidin-7-one (**270**) was performed (Mohammed et al., 2015). Metal-free condensation of aromatic ketones **269** with azole **268** was induced by the molecular iodine (10% mol) with oxygen in DMSO at 110°C and resulted in the formation of the Schiff bases but not the oxidation of the acetophenone to the 2-oxo-2-arylacetaldehyde that was observed in case of 110% I_2_ excess. Continuous heating promoted further intermolecular condensation with the formation of 1,6-dihydro-7*H*-pyrazolo[4,3-*d*]pyrimidin-7-one **270**. Attempts to expand the range of the substrates have shown that aliphatic ketones couldn't be used as the reagents; N-substituted amides also did not undergo the condensation under such conditions. Application of the K_2_S_2_O_8_ in acetonitrile–water mixture (1:1) at the room temperature (Hudwekar et al., 2017) allowed to apply the procedure not only for carbonyl compounds, but also for benzylamines or benzyl alcohol via their *in situ* oxidation. The formation of 1,5-dihydro-4*H*-pyrazolo[3,4-*d*]pyrimidin-4-ones **273** was also observed in the reaction of 5-amino-1*H*-pyrazole-5-carboxamide **271** and methyl phenylacetate (**272**) in EtOH-EtONa (Tintori et al., 2015; Figure 21).

The construction of the azoloazine ring may be performed by using other types of reactive groups in the β-position to the amino group of aminoazole. For example, 5-amino-1*H*-pyrazole-4-carbonitrile (**274**) reacted with formamide (**275**) under microwave irradiation at 200°C with the formation of 1*H*-pyrazolo[3,4-*d*]pyrimidin-4-amine (**276**) (Todorovic et al., 2011). The condensation of the benzamidine **278** proceeded in a similar way (Makarov et al., 2015; Figure 21).

**Figure 22:**
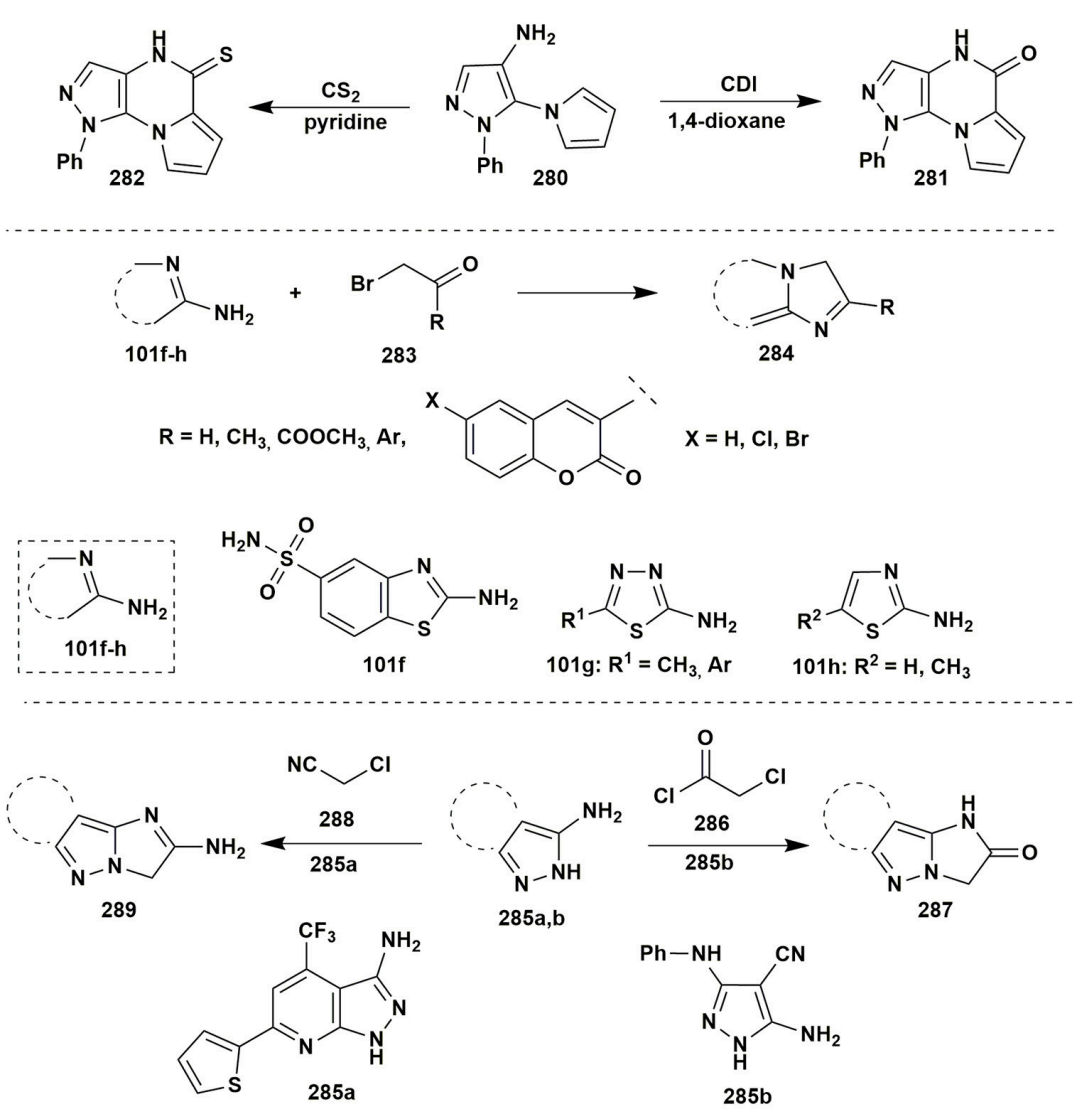
Other examples of two-component reactions involving aminoazles.

An interesting result was obtained in the condensation of 1-phenyl-5-(1*H*-pyrrol-1-yl)-1*H*-pyrazol-4-amine (**280**) with CDI in 1,4-dioxane or with CS_2_ in pyridine under refluxing that gave pyrazolo[4,3-*e*]pyrrolo[1,2-*a*]pyrazine systems **281**, **282** (Farghaly and El-Kashef, 2011; Figure 22).

Two component heterocyclization of aminoazoles **101f–h** and phenacyl bromide **283** or its aliphatic analogs was reported in numerous publications as a simple way to synthesize fused imidazoles **284**: imidazolo[2,1-*b*]benzothiazole (Chandak et al., 2013), imidazo[2,1-*b*][1,3,4]thiadiazole (Copin et al., 2016) were obtained under refluxing in EtOH, CCl_4_, MeCN. It should be noted, that the formation of uncyclized reaction products is often observed due to the protonation of the exocyclic amino group to form hydrobromic acid salts (Kamal et al., 2010; Figure 22).

2-Chloroacetyl chloride (**286**) in glacial acetic acid and 2-chloroacetonitrile (**288**) in DMF-KOH also could be applied in such type of heterocyclizations with the formation of 3,5-dihydro-4*H*-imidazol-4-ones **287** and 4*H*-imidazol-5-amines **289** (Rateb, 2014; Soliman et al., 2014; Figure 22).

**Figure 23:**
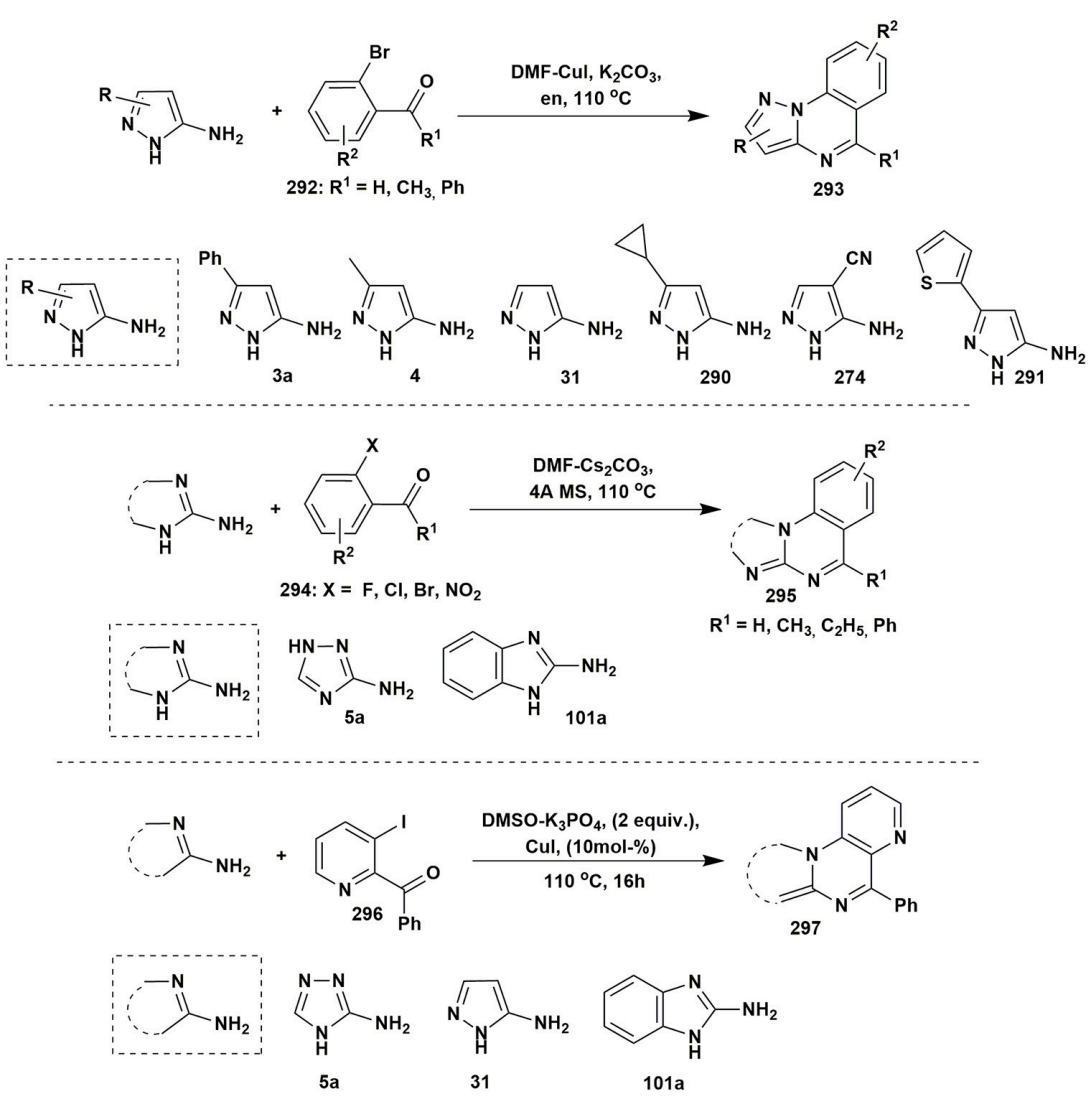
Reactions of aminoazoles and β-halogen containing aromatic carbonyl compounds.

Reactions of aminoazoles and β-halogen containing carbonyl compounds were reported as a way for the synthesis of angular fused heterocyclic systems. The copper catalyzed synthesis of the pyrazolo[1,5-*a*]quinazolines **293** was published by Gao et al. The most promising results were obtained in the system DMF-CuI-K_2_CO_3_ while other types of alkali media gave the worth results. It was found, that the application of the CuI, additionally stabilized by ethylenediamine was the most effective (Gao et al., 2014; Figure 23).

On the other hand, Nue et al. showed that 2-F and 2-NO_2_-derivatives **294** may be also applied in such reactions. Simple heating of the starting reagents in dry DMF-Cs_2_CO_3_ yielded angular heterocycles **295**. Unlike previous authors, application of the K_2_CO_3_ instead of Cs_2_CO_3_ gave worth results. The absence of the molecular sieves decreased the yield of the target [1,2,4]triazolo[1,5-*a*]quinazoline **295** from 84 to 74%. Monitoring the reaction mixture by HRMS showed the presence of the Schiff base, that could be one of the intermediates of the heterocyclization (Fang et al., 2014; Niu et al., 2014; Figure 23).

Hedidi et al. reported the copper catalyzed synthesis of pyrido[2,3-*e*]pyrimidines **297** (Hedidi et al., 2017). The attempts to obtain the target compounds via simple heating of the reagents **5a, 31, 101a**, and **296** with Cs_2_CO_3_ in DMF, as it had been reported in Niu et al. (2014), were unsuccessful while application of the procedure reported by Gao et al. (2014) allowed to fix their traces. The best results were observed in the system DMSO-CuI-K_3_PO_4_ without any ligand (Figure 23).

**Figure 24:**
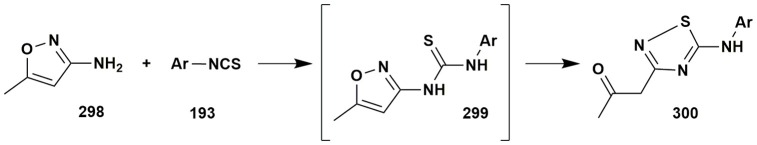
Boulton–Katritzky rearrangement in the reactions of 3-amino-5-methylisoxazole with arylisothiocyanates.

3-Amino-5-methylisoxazole was sometimes considered as a 1,3-binucleophile reacting with the preservation of the isoxazole moiety. However, in some cases establishing structures of the compounds synthesized without X-Ray data was not sufficient (Rajanarendar et al., 2016; Diyanatizadeh and Yavari, 2017). Sometimes the structures of final compounds were assigned similarly with the pyrazole-containing compounds, which in our opinion may be incorrect due to the possibility of isoxazole ring opening. For instance, the condensations of 3-amino-5-methylisoxazole (**298**) with arylisothiocyanate **193** with further Boulton—Katritzky rearrangement result in the formation of the 1,2,4-thiadiazoles **300** (Pokhodylo and Shyyka, 2014; Proshin et al., 2014; Figure 24).

Thus, two component reactions involving aminoazoles and substrates of various origins allow forming diverse azoloazine, azinoazine and other heterocyclic systems. The substrates for condensations are not limited to 1,3-dielectrophiles or carbonyl compounds although they constitute the overwhelming majority of typical reagents.

### Click chemistry concerning azoles and aminoazoles

Click chemistry, by B. Sharpless definition (Kolb et al., 2001), describes reactions that are wide in scope, suitable for most substrates, stereospecific, have high yields and low amount of side products, the latter can be removed without application of chromatography methods. The process itself needs to be conducted in mild conditions, the reactants–to be readily available, the solvent–to be easily removed or absent, and the product–to be effortlessly separated from the reaction mixture. The concept of click chemistry perfectly goes along with the principles of green chemistry and with diversity oriented synthesis due to the possibility to build different types of molecular skeleton and may be used for synthesis and further modification of aminoazoles as well.

Talking about click chemistry, azide-alkyne cycloaddition is always the first thought, but the authors of the term (Kolb et al., 2001) also include to the massive of click reactions the following:

- [3 + 2], [4 + 2] and [4 + 1] cycloadditions, Diels-Alder reaction, in particular;
- Nucleophilic addition, oxirane and aziridine ring opening;
- Some heterocyclization reactions
- Reactions of carbonyl compounds: azomethine derivatives formation, epoxidation, Michael reaction.

The most obvious reason for the development of this field of study is the minimization of efforts for obtaining the final product by means of resource economy. The advantages of click chemistry are useful for the purposes of pharmacology and medicinal chemistry (Choi et al., 2006; He et al., 2016). Wastelessness and bioorthogonality of this reaction type promoted its implementation into medicinal chemistry and caused, for example, development of new molecules for contrast identification of cancer cells (Lee et al., 2014), RNA and DNA molecules, proteins (Shieh et al., 2015), etc.

Although, as it was mentioned, the most popular first thought about click-chemistry is the Cu(I)-assisted synthesis of 1,2,3-triazoles, in this part of the review we will focus on the procedures with different starting reactants rather than publications discussing new catalysts for the reaction of azide and alkyne.

**Figure 25:**
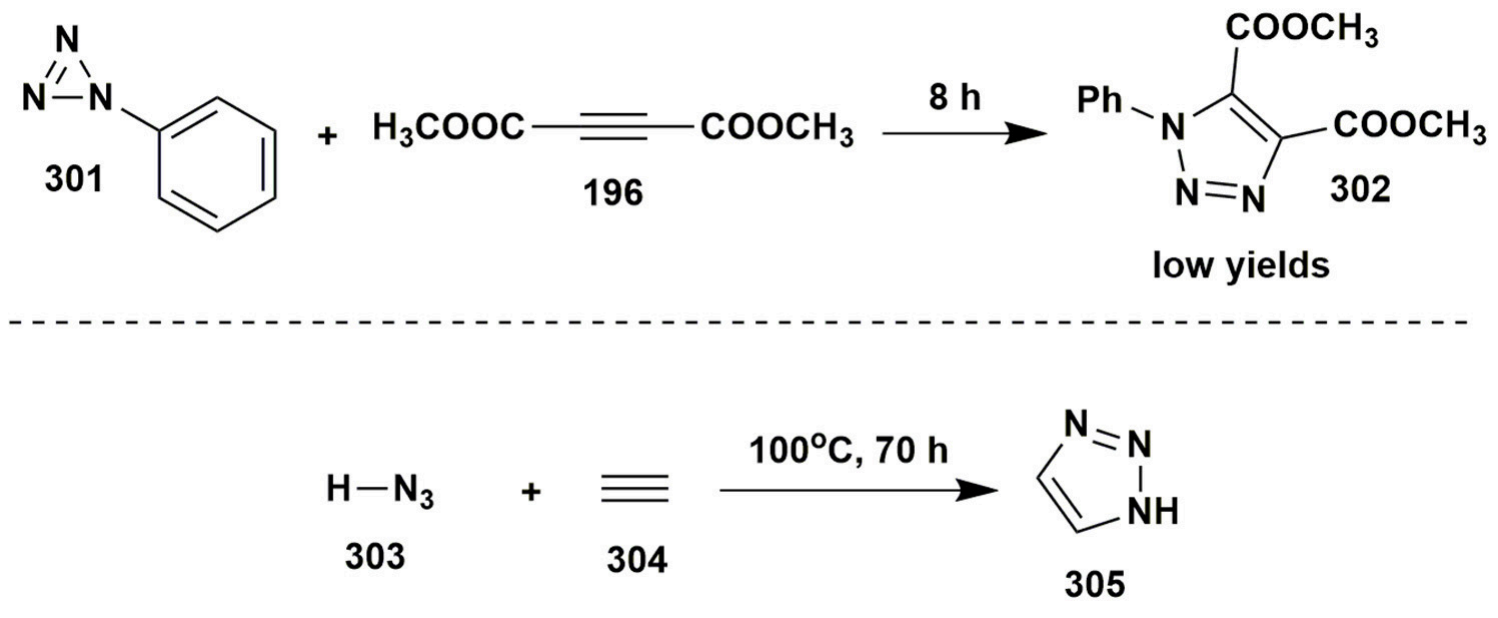
First attempts for 1,2,3-triazole moiety synthesis.

The pre-click triazole-forming cycloaddition reactions were well-known in the nineteenth century, but were very inconvenient as they required long-term heating in closed vessels, thus, could be in no way characterized as “click” reactions. As an example, one of those methods (Michael, 1893) included addition of 2-phenyl-*2H*-triazirine (**301**) to dimethyl acetylenedicarboxylate (**195**) in molten form and the product **302** was formed in low yields (Figure 25).

The first azide-alkyne reaction involved transformation of hydrogen azide (**303**) and acetylene (**304**) in ethanol-acetone mixture in a closed vessel for 70 h (Dimroth and Fester, 1910). Such unfriendly reaction conditions closed the door to 1,2,3-triazole (**305**) synthesis and research of the properties of these heterocycles for decades (Figure 25). It should be noted, that the original paper (Dimroth and Fester, 1910) can be hardly found in the journal, though there exists a plenty of references in many papers and theses.

Unarguably, the simplest method to obtain 1,2,3-triazole fragment is copper-catalyzed 1,3-dipolar azide-alkyne cycloaddition (CuAAC), firstly described by Meldal (Tornøe et al., 2002) and Sharpless (Rostovtsev et al., 2002) groups. Its mechanism was studied and published by Worrell et al. (2013), and mechanistic data was thoroughly reviewed by Berg and Straub (2013). Needless to say, CuAAC is highly progressing and, thus, popular object of research, described in a great number of papers and reviews (Hein and Fokin, 2010; Berg and Straub, 2013), and a plenty of other publications are devoted to this reaction in different subtopics, including solid-phase (Castro et al., 2016), green (Shirame and Bhosale, 2018), solvent-free (Tireli et al., 2017) syntheses.

Cu(I) only shows its great catalytic activity with terminal alkynes as reactants (Liang and Astruc, 2011). For non-terminal alkynes catalysts on the basis of platinum metals are known, such as ruthenium (Boren et al., 2008; Johansson et al., 2016) and palladium (Kamijo et al., 2002). Metal-free azide-alkyne cycloadditions and photoclick reactions are also known and intensively studied (Rodríguez-Rodríguez et al., 2015; Singh et al., 2016; Jalani et al., 2017).

**Figure 26:**
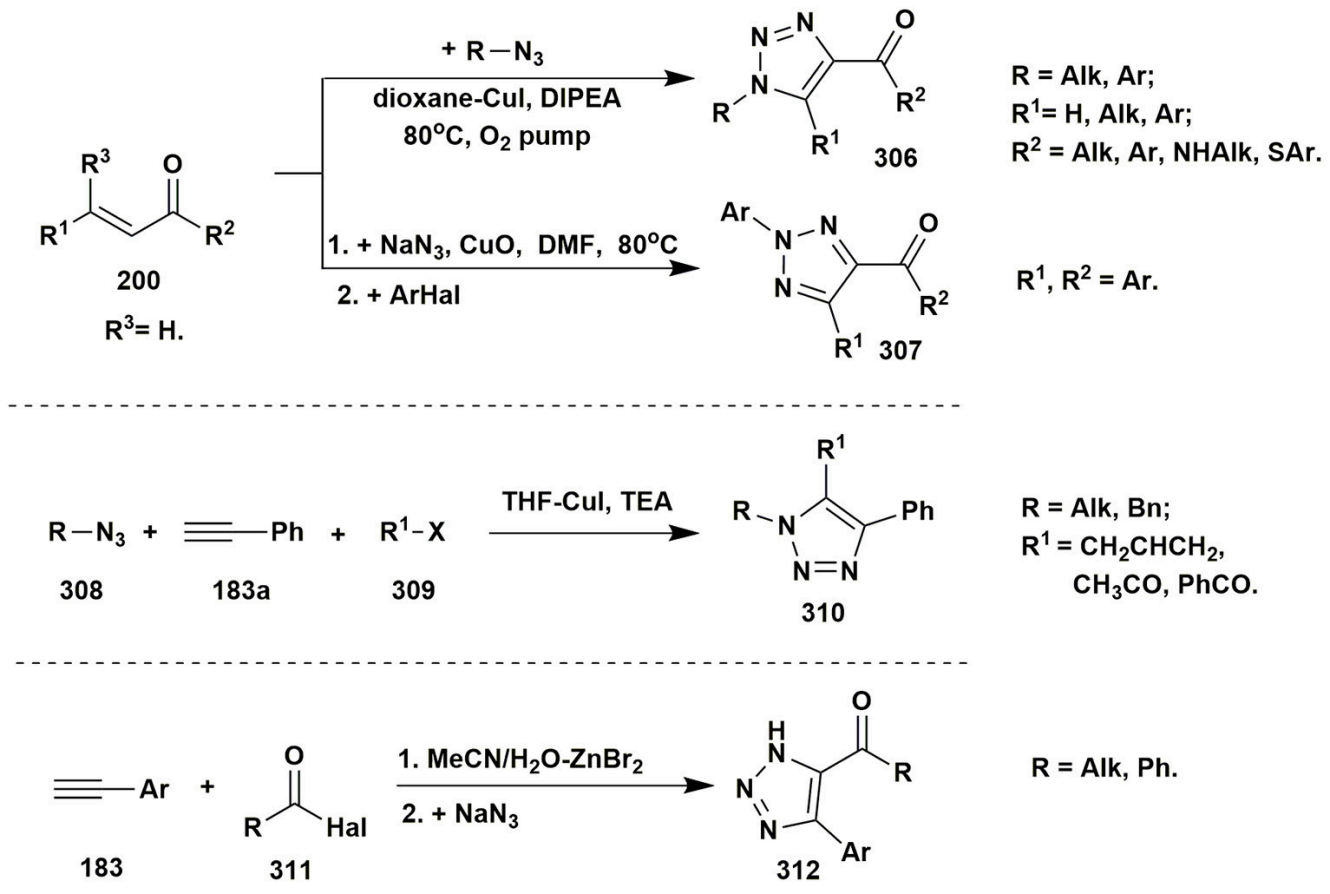
Azide-based cycloaddition in synthesis of 4-acyl-1,2,3-triazoles.

Regardless of the novelty of CuAAC method it has quickly become the most popular procedure for the synthesis of 1,2,3-triazole derivatives. Variations were also invented for preparation of these heterocycles from alkenes. For instance, Janreddy et al. (2013) report successful cycloaddition of organic azides **308** to α,β-unsaturated ketones **200**, such as vinyl ketones and chalcones, in an oxidative atmosphere of pure oxygen. As an oxidant CuO can also be employed, as a more convenient reagent than gaseous oxygen (Figure 26). In another paper, the triazole ring was also arylated by aryl halides without separation to obtain triazoles **307** (Zhang et al., 2012).

1,2,3-Triazole fragments were also obtained by multicomponent ways. For example, Wu et al. (2005) reported reaction of organic azide **308** and terminal alkyne **183** in the presence of Cu(I) salts and following cleavage of C-Cu bond in an Ullmann-like reaction by an alkylating agent **309**, which resulted in 1,4,5-substituted triazole **310** (Figure 26).

Another multicomponent approach (Figure 26) included consecutive reaction of terminal alkyne **183** with acyl halide **311**, and then with sodium azide with application of zinc bromide, which served as a catalyst for both steps of the process (Keivanloo et al., 2013).

Another type of click reactions–syntheses of tetrazoles–were carried out even more than a century ago (Pinner, 1894; Dimroth and Merzbacher, 1910), but those tries took a lot of time and energy, requiring 40 h-long refluxing in ethanol, heating in a sealed vial under high pressure etc. Such methods were reviewed by Benson (1947).

Only the second half of XX century contributed easier and faster procedure of tetrazole fragment formation. An end-of-the-century review was published in 1994 (Wittenberger, 1994) and included latest advances in synthesis, functionalization and applications of these heterocycles.

**Figure 27:**
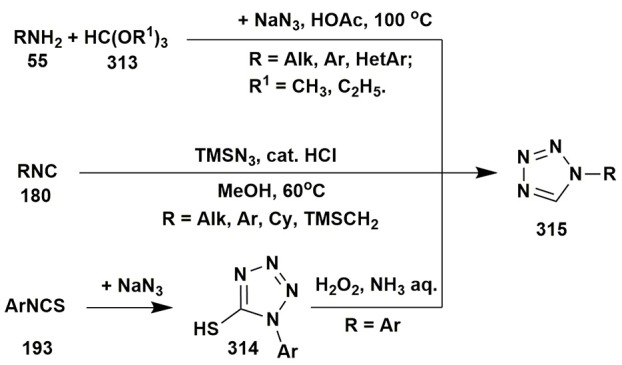
Syntheses of N-substituted tetrazoles.

Three-component reactions of primary amines **55** of various origin, including aminoazoles, orthoformic ester **313** and sodium azide leading to 1-substituted tetrazoles **315** (Figure 27) was thoroughly studied by Gaponik group and their followers (Gaponik et al., 1985, 1990; Voitekhovich et al., 2005, 2013). One of the latest and most complete reviews was presented in Grigoriev et al. (2017).

Isonitriles **180** were shown to react with trimethylsilylazide in the presence of hydrochloric acid (Jin et al., 2004), forming 1-substituted tetrazoles **315**. Arylisothiocyanates **193** in the reaction with sodium azide form 5-thiotetrazoles **314**, which could be oxidized to 1-aryltetrazoles **315** by hydrogen peroxide, chromium trioxide and other oxidizers (Joule and Mills, 2010; Figure 27).

**Figure 28:**
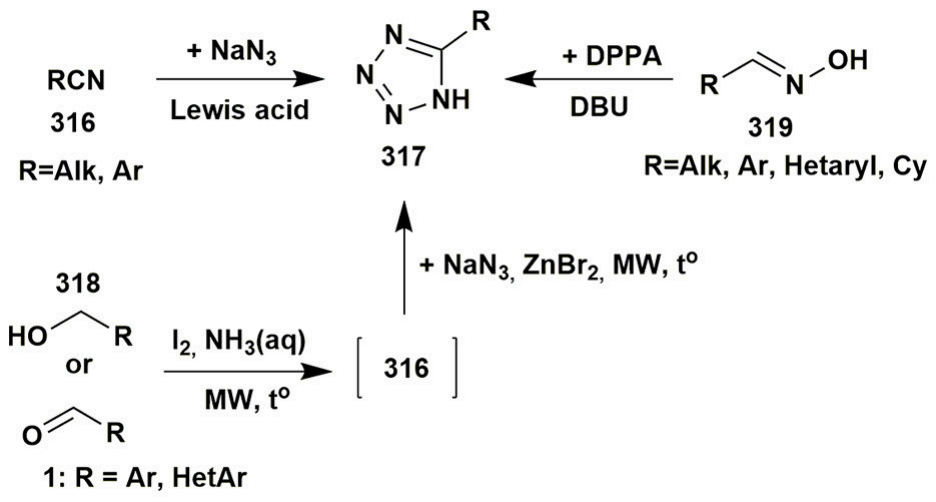
Syntheses of C-substituted tetrazoles.

Synthesis of C-substituted tetrazoles **317** is more widely studied, and the number of methods for their preparation is larger. A Lewis acid-assisted reaction of organic nitriles **316** and sodium azide is probably the most popular (Figure 28). Yields were reported to be as high as 90% for benzonitriles in the presence of ZrOCl_2_, but using zinc salts became a classic procedure (Galante and Somerville, 1996).

Nitriles **316**, reactive toward [3 + 2]-cycloaddition reactions with azide, can be formed *in situ* from primary alcohols **318** or aldehydes **1** by oxidation with iodine in aqueous ammonia under microwave irradiation (Shie and Fang, 2007; Figure 28). Tetrazoles **317** were reported to be separated in high yields in such procedure.

Diphenylphosphorazidate (DPPA) served as a reactant in conversion of aldoximes **319** to 5-substituted tetrazoles **317** (Figure 28), making the publication (Ishihara et al., 2018) different from other methods employing aldoximes as initial compounds by the relative safety of the procedure, which excludes explosive azide sources and heavy metals.

5-Aryl-1-substituted tetrazoles **321** can also be obtained via click reactions. The method (Kaim et al., 2011) suggests mixing of isonitriles **180** with bromine and sodium azide in acetonitrile; arylboric acid with Suzuki catalysts are introduced to the reaction mixture without separation of an intermediate product **320**.

**Figure 29:**
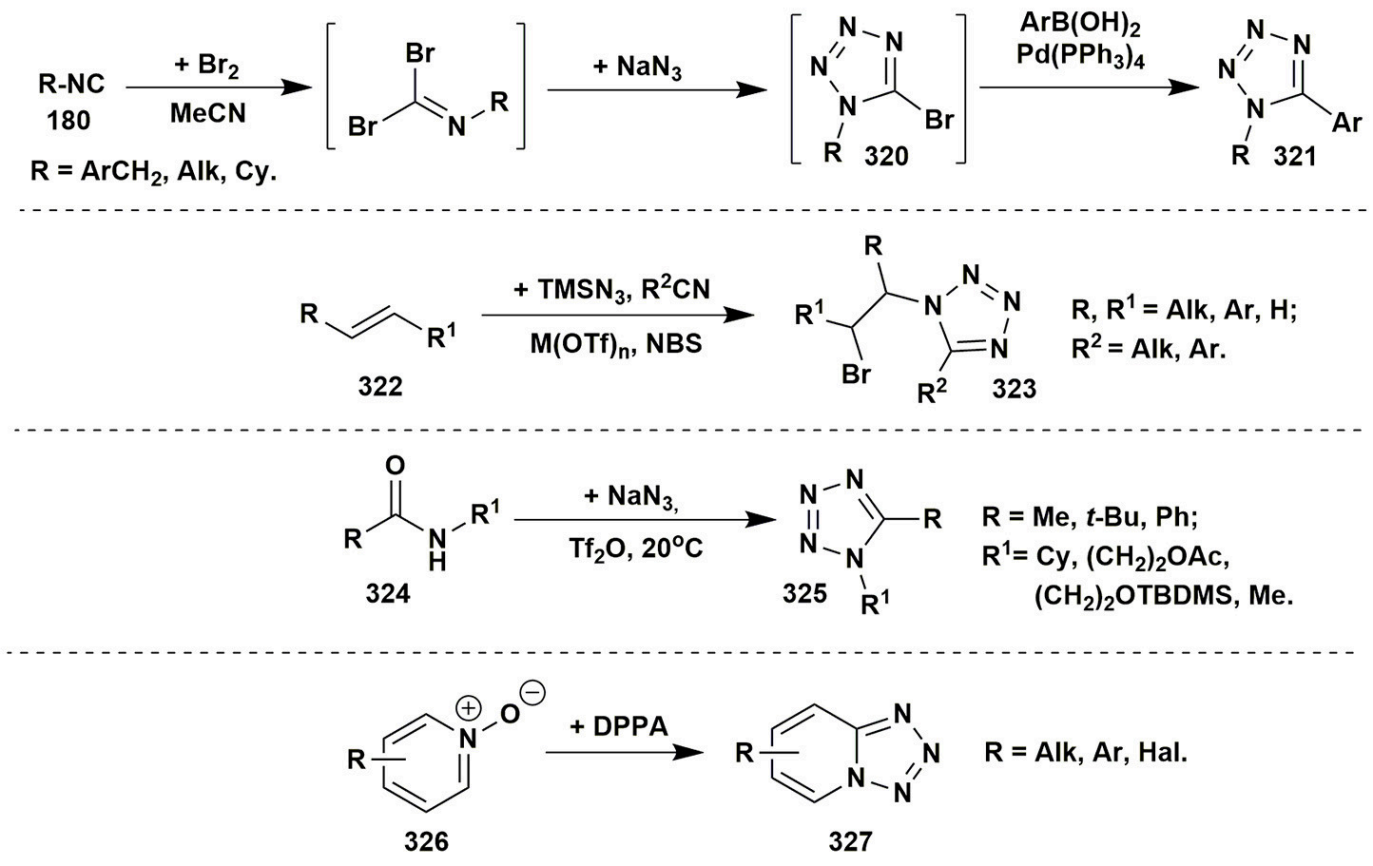
Syntheses of disubstituted tetrazoles.

Other method of preparation of 1,5-disubstituted tetrazoles **323** can include one-pot transformation of an alkene **322**, *N*-bromosuccinimide, nitriles and trimethylsilane azide, catalyzed by triflates and is reported by Hajra et al. (2007). Yields appeared to be higher when zinc triflate was used (Figure 29).

Methods of preparation of tetrazole **325** starting from carboxamides **324** are also known. In this case, amide group needs to be activated, for example, by trifluorosulfonic acid anhydride (Thomas, 1993) and then [3 + 2]-cycloaddition of azide proceeds (Figure 29).

Tetrazolopyridine **327** synthesis was described by Keith (2006). Pyridine N-oxides **326** were allowed to react with phosphorylazides in hot pyridine as a solvent. Various phosphorylazides were tested, but diphenylphosphorazidate (DPPA) proved to be the most convenient source of azide group (Figure 29).

**Figure 30:**
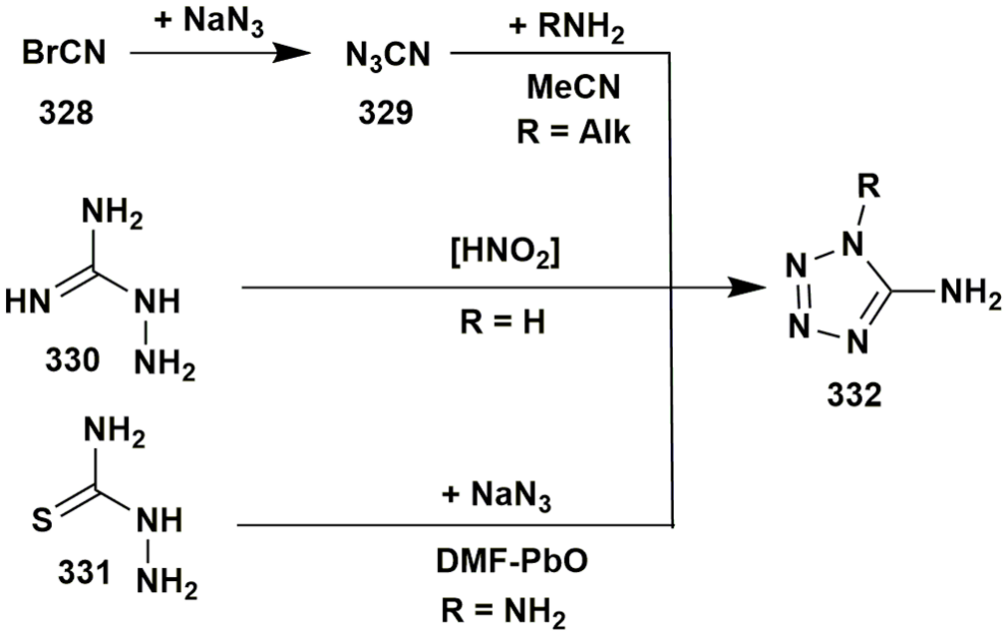
Syntheses of 5-aminotetrazoles.

1-Alkyl-5-aminotetrazoles **332** can be obtained by reaction of cyanazide **329** and primary amines, which form intermediate amidoylazide with subsequent cyclization in acetonitrile-water mixture (Joo and Shreeve, 2008; Figure 30).

5-Aminotetrazole (**5b**) (**332** R = H) was synthesized by nitrosation of aminoguanidine (**330**) and following heterocyclization of the intermediate with high yields (Kurzer and Godfrey, 1963; Figure 30). 5-Molar nitric acid needs to be considered as a hazard in this synthesis.

1,5-Diaminotetrazole (**332**) (R = NH_2_) was formed by heating thiosemicarbazide (**331**) suspension in dry DMF with ammonium chloride, lead(II) oxide and sodium azide (Gaponik and Karavai, 1984). Only freshly obtained red modification of PbO proved to be useful in this reaction (Figure 30).

## Conclusions

In summary, comprehensive analysis of the literature concerning the topic of the present review demonstrates that reactions involving aminoazoles as key reagents possess a high potential for diversity-oriented synthesis and open up effective and convenient pathways to numerous types of final heterocyclic compounds. Classical two-component and stage-by-stage procedures as well as multicomponent reactions of aminoazoles allow to synthesize diverse five-, six- and seven-membered heterocycles using a limited set of reagents the most common of which are α,β-unsaturated carbonyl and carboxyl compounds, cyclic and non-cyclic CH-acids, aldehydes, ketones and diketones of different origin. Additional benefits may be obtained by application of such innovative approaches as microwave- and ultrasonic-assisted organic synthesis, methods of click-chemistry, using special catalysts etc. The compounds synthesized from aminoazoles are useful as building-blocks for further construction of complex heterocyclic systems, as promising objects of medicinal-oriented chemistry to search for the novel drug-like substances and as components of functional materials.

## Author contributions

YS and SD collected most publications related to this review article and sorted them. MM analyzed the literature and wrote the chapter about multicomponent reactions. AM analyzed the literature and wrote the chapter about two-component reactions. IZ analyzed the literature and wrote the chapter about click-chemistry. VC developed the concept of the review, co-wrote and corrected the manuscript.

### Conflict of interest statement

The authors declare that the research was conducted in the absence of any commercial or financial relationships that could be construed as a potential conflict of interest.
